# Quantitative Methods to Monitor RNA Biomarkers in Myotonic Dystrophy

**DOI:** 10.1038/s41598-018-24156-x

**Published:** 2018-04-12

**Authors:** Marzena Wojciechowska, Krzysztof Sobczak, Piotr Kozlowski, Saam Sedehizadeh, Agnieszka Wojtkowiak-Szlachcic, Karol Czubak, Robert Markus, Anna Lusakowska, Anna Kaminska, J. David Brook

**Affiliations:** 1 University of Nottingham, Queen’s Medical Centre, School of Life Sciences, Nottingham, NG7 2UH United Kingdom; 2 Institute of Bioorganic Chemistry, Polish Academy of Sciences, Department of Molecular Genetics, Poznan, 61-704 Poland; 3 Institute of Molecular Biology and Biotechnology, Adam Mickiewicz University, Department of Gene Expression, Poznan, 60-614 Poland; 4 Medical University of Warsaw, Department of Neurology, Warsaw, 02-097 Poland

## Abstract

Myotonic dystrophy type 1 (DM1) and type 2 (DM2) are human neuromuscular disorders associated with mutations of simple repetitive sequences in affected genes. The abnormal expansion of CTG repeats in the 3′-UTR of the *DMPK* gene elicits DM1, whereas elongated CCTG repeats in intron 1 of *ZNF9*/*CNBP* triggers DM2. Pathogenesis of both disorders is manifested by nuclear retention of expanded repeat-containing RNAs and aberrant alternative splicing. The precise determination of absolute numbers of mutant RNA molecules is important for a better understanding of disease complexity and for accurate evaluation of the efficacy of therapeutic drugs. We present two quantitative methods, Multiplex Ligation-Dependent Probe Amplification and droplet digital PCR, for studying the mutant *DMPK* transcript (*DMPK*^exp^RNA) and the aberrant alternative splicing in DM1 and DM2 human tissues and cells. We demonstrate that in DM1, the *DMPK*^exp^RNA is detected in higher copy number than its normal counterpart. Moreover, the absolute number of the mutant transcript indicates its low abundance with only a few copies per cell in DM1 fibroblasts. Most importantly, in conjunction with fluorescence *in-situ* hybridization experiments, our results suggest that in DM1 fibroblasts, the vast majority of nuclear RNA foci consist of a few molecules of *DMPK*^exp^RNA.

## Introduction

Myotonic dystrophy type 1 (DM1) and type 2 (DM2) are adult onset muscular dystrophies leading to disability and shortened lifespan. Both belong to a larger group of human disorders associated with mutational expansions of simple repetitive sequences within specific genes. The mutation that causes DM1 results from the expansion of CTG repeats in the 3′-UTR of the *DMPK* gene; whereas DM2 is associated with intronic elongation of CCTG repeats in *ZNF9*/*CNBP* gene^1,2^. Pathogenesis of both diseases is mediated by a toxic RNA *gain-of-function* mechanism manifested by nuclear retention of expanded CUG- and CCUG-harboring RNAs which aggregate within nucleoprotein foci^3^. Their adverse effects are mediated through sequestration of various proteins including the *muscleblind*-like (MBNL) family of splicing factors. These proteins are functionally depleted by the expanded repeats in DM1 and DM2 which leads to abnormalities in many pathways of RNA metabolism including alternative splicing, a molecular hallmark of DM^4–8^.

In DM adult skeletal muscle abnormal expression of embryonic splicing isoforms has been reported for a few hundred genes^4^. Given the importance of alternative splicing as one of the biomarkers of disease severity and therapeutic response, accurate quantification of splicing variants could facilitate their refinement for clinical applications. Specific oligonucleotide-based microarrays and next-generation sequencing have been used for profiling and identification of many aberrantly spliced transcripts. However, their routine application for precise quantification remains limited due to low quantitative parameters of these methods, restriction to highly expressed genes, high costs and the large amount of RNA required. On the other hand, traditional analysis of splicing isoforms via primer design, PCR and agarose gel visualization of PCR products shows low sensitivity, low repeatability and reproducibility between different laboratories. Thus, new methods of alternative splicing analysis enabling robust and reliable quantification of splicing isoforms are needed for many aspects of DM research, and the first aim of this study is to optimize such new approaches.

Understanding the biology of mutant *DMPK* transcripts (*DMPK*^exp^RNA) associated with DM1 pathogenesis involves quantification of the toxic molecules per cell. Thus, selection of the most sensitive and reliable methodologies is essential to compute the absolute number of disease-associated molecules. The most recent study measuring the abundance of endogenous *DMPK* transcripts revealed that in human DM1 myoblasts both normal (*DMPK*^norm^RNA) and mutant mRNAs are low-abundance species^9^. Several lines of evidence including Northern blotting, quantitative RT-PCR and RNA-sequencing showed that there are between one and a few dozen *DMPK* mRNAs per cell, half of which contain the repeat expansion. In a similar study looking at the abundance and processing of an antisense transcript across the *DMPK* repeat expansion, only a handful of repeat containing antisense transcripts were computed per cell^10^. Herein, we calculate concentration of *DMPK* mRNA in human DM1 samples using a novel ddPCR approach which is an absolute quantification method. Optimization of this technique to develop a further biomarker of DM1 is the second aim of this current work.

Two methods based on Multiplex Ligation-Dependent Probe Amplification (MLPA) and droplet digital PCR (ddPCR) were used herein for quantification of RNA biomarkers of DM pathogenesis, including aberrant alternative splicing and *DMPK*^exp^RNA copy number. MLPA and ddPCR are reliable dosage quantification methods and various applications include large mutation detection, genotyping of copy number variants, evaluation of NGS library exome enrichment efficiency, methylation analysis, and high-sensitivity gene expression studies^11–18^. MLPA is a multiplex method simultaneously utilizing multiple probes that allows quantification of different target sequences. Each probe is composed of two half-probes that hybridize to directly adjacent target sequences. Only half-probes specifically recognizing their targets undergo subsequent ligation and dosage-dependent amplification with a pair of universal primers. The MLPA products consisting of a mixture of probe-specific PCR fragments are separated by capillary electrophoresis and quantified. The normalized signals from MLPA probes are proportional to the dosage of their targets.

ddPCR is a highly quantitative and precise method which enables absolute nucleic acid measurement based on the partitioning of individual molecules into many low-volume droplet reactions at limiting dilution, resulting in one or zero molecules in most reactions in individual droplets^17,19,20^. After endpoint PCR, the starting concentration of template is determined by Poisson statistical analysis of the number of positive (containing amplified target) and negative (no amplified target detected) reactions. The creation of several thousands of droplets in one reaction means that a single sample can generate several thousands of data points rather than a single result, bringing the power of statistical analysis of ddPCR into practical application. Thus, the digital PCR procedure displays many potential advantages over real-time PCR, including the capability to obtain absolute quantification without external references and robustness to variation in PCR efficiency.

Knowing the precise number of disease-causing molecules is of high importance since it will allow a better understanding of DM pathology and successful drug discovery. Herein, we show that a robust and reliable quantification of RNA biomarkers of DM pathogenesis can be achieved by using two medium-throughput methods based on MLPA and ddPCR. Both methods have proven to generate highly reproducible results allowing simultaneous quantification of splicing isoforms of many genes that are aberrantly spliced in DM. In addition, we provide a novel quantitative approach for computing the absolute number of *DMPK* transcripts in copies per cells using ddPCR. Most importantly, in conjunction with fluorescence *in-situ* hybridization experiments, our results suggest that in DM1 fibroblasts, the vast majority of nuclear RNA foci consist of a few molecules of mutant *DMPK* transcripts.

## Results

### Design of MLPA and ddPCR Assays for Quantitative Analysis of Alternative Splicing Profiles

**Figure 1:**
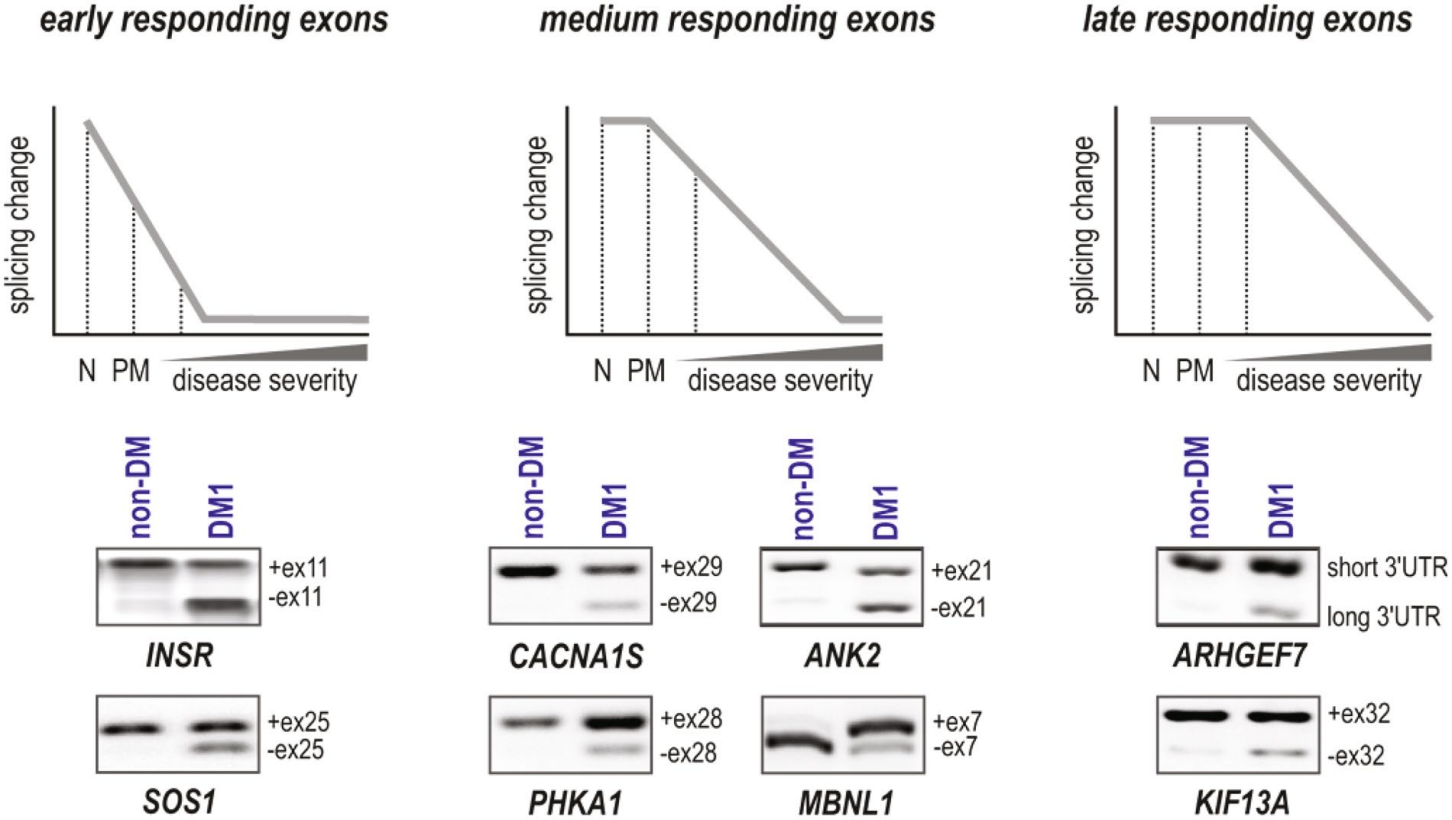
Targets Selected for Analysis of Aberrant Alternative Splicing. Three categories of alternative exons selected are shown: early responding exons (*INSR* E11 and *SOS1* E25); medium responding exons (*CACNA1S* E29, *ANK2* E21, *PHKA* E28 and *MBNL1* E7), and late responding exons (*KIF13A* E32 and *ARHGEF7* 3′UTR). N, normal samples; PM, samples from pre-mutation carriers and DM patients with increasing disease severity. The agarose gel separation of representative RT-PCR products for alternative exon inclusion and exclusion in non-DM (sample 6A, female, tibialis anterior) and DM1 (sample 3A; adult onset, male, tibialis anterior; 390 CTG repeats) is shown.

Aberrant alternative splicing is a hallmark of DM pathogenesis and precise calculation of splicing isoforms remains challenging. The first aim of this work was to design sensitive, reproducible and highly quantitative methods to monitor alternative splicing changes in skeletal muscles of DM1 and DM2 patients which may serve as accurate biomarker of disease progression that could be used in clinical trials. For this purpose we selected eight alternative exons whose splicing profiles correlate well with disease severity measured as a loss of muscle strength^4^. We divided these exons into three categories according to the timing of alternative splicing origin (Fig. 1). The first category (early responding exons) comprised exons in two genes: *INSR* E11 and *SOS1* E25, with decreased inclusion observed in all DM1 patients, including pre-mutation *DMPK* allele carriers. The second category (medium responding exons) consisted exons of four genes: *CACNA1S* E29, *ANK2* E21, *PHKA* E28 and *MBNL1* E7 with inclusion rates changed in all affected DM1 patients showing high correlation with disease severity. The third category (late responding exons) comprised exons in two genes: *KIF13A* E32 and *ARHGEF7* 3′UTR which are altered strongly in the most affected DM1 patients. We used several criteria to select these eight exons: (i) a high correlation between splicing changes and muscle strength index in DM1 patients, (ii) a low variance of exon inclusion rate in the muscles of healthy individuals, (iii) constitutive exons flanking exons of interest not affected by alternative splicing, and (iv) a similar expression level of genes carrying analyzed exons to allow for their simultaneous analysis. Among the eight genes, *INSR* E11 was the only exception where criteria (i) and (ii) were not fulfilled.

**Figure 2:**
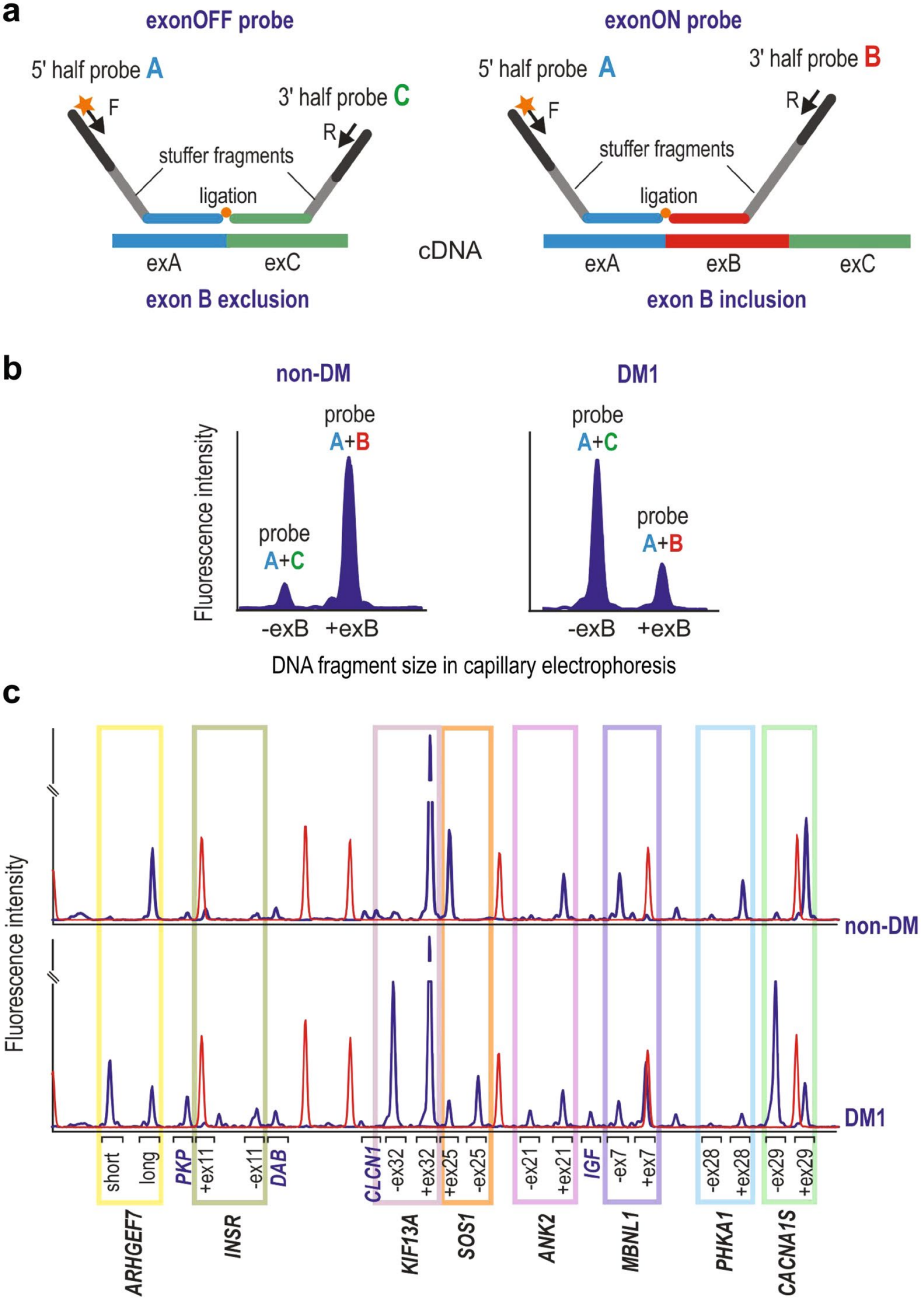
MLPA Assays Designed. (**a**) Schematic representation of the ex-OFF probe (left panel) and the ex-ON probe (right panel) specifically hybridized to the cDNA of a transcript, respectively, without and with the alternative exon B. Exons A and C are constitutive exons flanking the alternative exon B. Each of the exon-specific probes is composed of a 5′ half-probe and a 3′ half-probe (5′ half-probe is shared by both ex-OFF and ex-ON probes). Each half-probe is composed of a target-specific sequence (indicated by a color corresponding to targeted exon), stuffer sequence (gray), and sequence specific to either F (labeled) or R universal primers. Note that stuffer sequence in 3′ half-probe is different in size in the ex-OFF and ex-ON probes allowing differentiating the signal of probes in capillary electrophoresis. (**b**) Comparison of ssMLPA signals (electropherogram peaks) of the hypothetical exon-specific pair of probes (shown in panel a) in non-DM and DM1 samples (note, different ratio of signals of ex-ON and ex-OFF probes in compared samples). The ratio of signals from both peaks was used to calculate the PSI value for each sample. (**c**) Representative ssMLPA results (electropherograms) of non-DM and DM1 samples. Blue peaks represent signals of MLPA probes used for indicated genes. The color rectangles indicate the pairs of exon-specific probes. The signals outside the rectangles represent control probes. Red peaks denote GS Liz600 DNA size standard.

Using novel methodologies based on counting data from single MLPA^21^ and ddPCR^17^ reactions we show that simultaneous and accurate quantitation of two splicing isoforms either exon inclusion (ex-ON) or exclusion (ex-OFF) can be achieved with both methods. First, for multiplex analysis of the exons, we designed a splicing-specific MLPA (ssMLPA) assay consisting of 16 MLPA probes (Supplementary Table S1). As shown in Fig. 2a, there are two probes designed for each of the alternatively-spliced exons: an ex-ON probe and an ex-OFF probe. Each pair of exon-specific probes shares one half that is specific for the exon preceding the alternative exon (flanking half-probe) and another half specific for either alternative or consecutive constitutive exon (exon-specific half-probe). Additionally, the exon-specific part differs by the length of a stuffer sequence which allows ex-ON and ex-OFF probes to be distinguished in capillary electrophoresis (Fig. 2b). Because the target sequences of the exon-specific half-probes are directly adjacent to the exon-exon boundaries their ligation occurs only in the presence of cDNA corresponding to the particular transcript variant. Additionally, the assay consists of four control probes specific for different non-alternatively-spliced control transcripts for signal normalization. After MLPA reaction the products were size separated by capillary electrophoresis (Fig. 2c). The fluorescent signal of each probe was normalized against the signal of control probes and then the ratios of signals of each pair of exon-specific probes (i.e. ex-ON and ex-OFF) were measured to calculate the percent of spliced-in (PSI) values.

**Figure 3:**
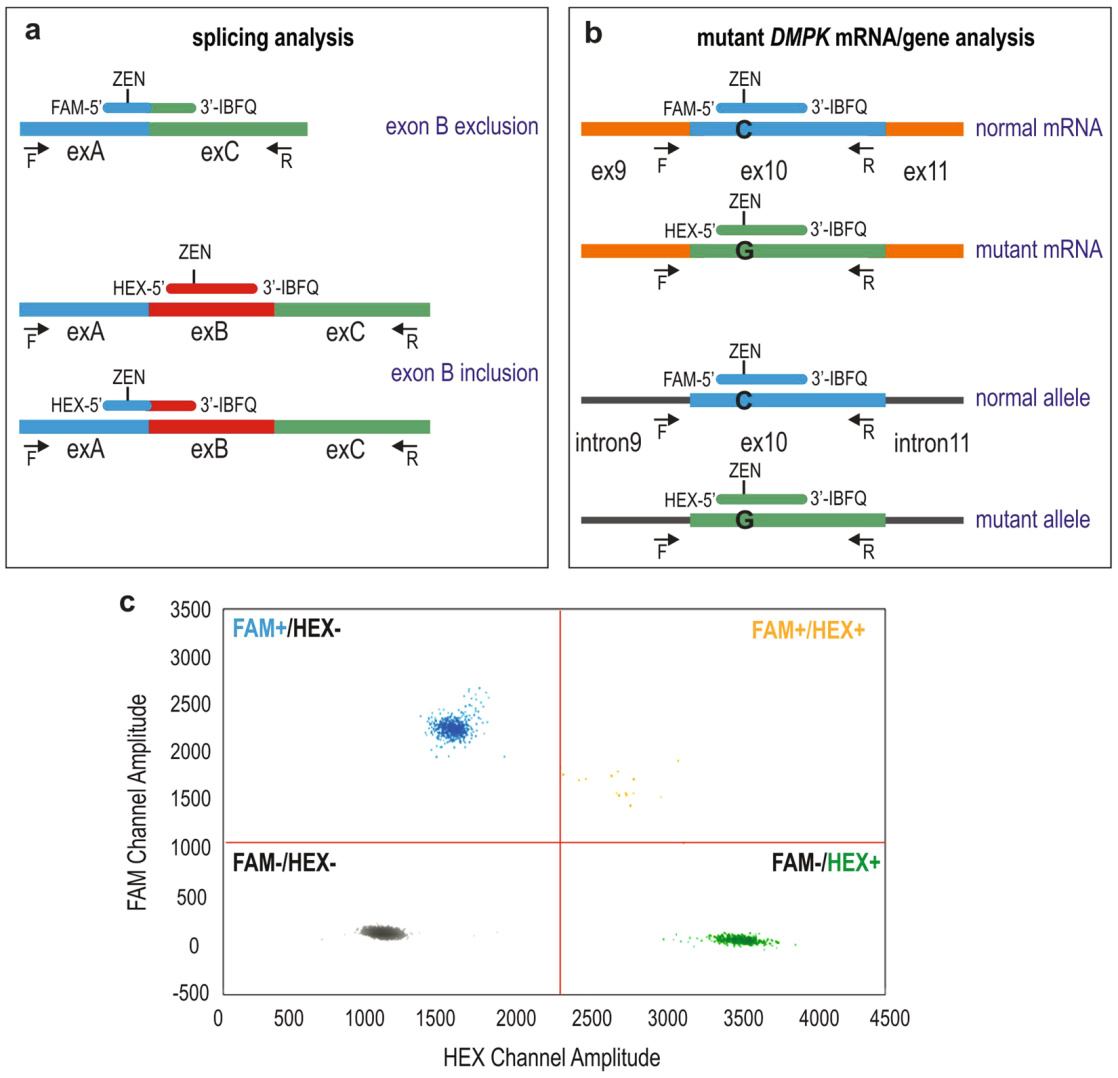
ddPCR Probes Used. (**a**) An alternative exon exclusion probe (FAM-labeled) and two alternative exon inclusion probes (HEX-labeled) used in alternative splicing assays; the probes have ZEN-Iowa Black as the dual-quencher. An alternative exon (exB) is indicated in red, and it’s flanking exons (exA and exC) in blue and green. Location of primers (F and R) is shown by arrows and ZEN probes are displayed in their binding exons. (**b**) Dual-quencher probes used in *DMPK* copy number quantification in cDNA (upper part) and gDNA (lower part). The rs527221 SNP in exon 10 of *DMPK* was used to distinguish normal-size and mutant alleles. Position of primers (F and R) is shown by arrows and probes are displayed in their binding sites. (**c**) Representative 2-dimensional scatter plot displaying droplet populations in separate clusters depending on their fluorescence amplitude following ddPCR for *CACNA1S* exon 29 (DM2 sample). FAM-positive droplets (FAM+/HEX−), HEX-positive (FAM−/HEX+), double-positive (FAM+/HEX+), and negative droplets (FAM−/HEX−) are shown.

For quantitative measurement of alternatively spliced products via ddPCR we designed two types of TaqMan hydrolysis probes (ZEN™ Double-Quenched Probes) specific to transcripts with (ex-ON) or without (ex-OFF) an alternative exon, and labeled at the 5′ end with either FAM or HEX fluorophore (Fig. 3a). Additionally, the probes are 3′-end modified with Iowa Black FQ quencher, and an internal ZEN quencher between the 9th and 10th bases from the 5′ end (Supplementary Table S2). For the purpose of assay reproducibility we designed two different sets of probes to measure ex-ON transcripts (Fig. 3a) (for details, please see Methods). ddPCR reactions consisted of ex-ON and ex-OFF probes and a set of primers were used for PCR amplification to generate products in independent droplets that were then analyzed via droplet reader (Bio-Rad). Eventually, depending on droplets’ fluorescence signals, they were clustered as FAM-, HEX- or double-positive, whereas droplets with no amplicons were considered negative (Fig. 3c). Using the assays outputs as copies/µl for FAM and HEX-labeled products from the Bio-Rad reader software, the PSI values were calculated for each alternative exon.

### Aberrant Alternative Splicing Analysis via MLPA and ddPCR in Human Skeletal Muscle

We compared two quantitative approaches to evaluate the extent of changes in alternative splicing in DM human skeletal muscles and in non-DM samples. We selected a representative group of samples derived from either distal (tibialis anterior) or proximal (quadriceps and biceps bronchi) muscles of DM1 (n = 7) and DM2 (n = 5) and control biopsies from the same muscles of non-DM individuals (n = 7) (Supplementary Table S3). The template in both approaches was cDNA obtained by reverse transcription reaction using random hexamers and 1 μg of total RNA. For each gene we calculated the percent spliced in (as described above) and normalized it to the average PSI value for non-DM samples, resulting in a delta PSI (|ΔPSI|) value for each DM sample.

**Figure 4:**
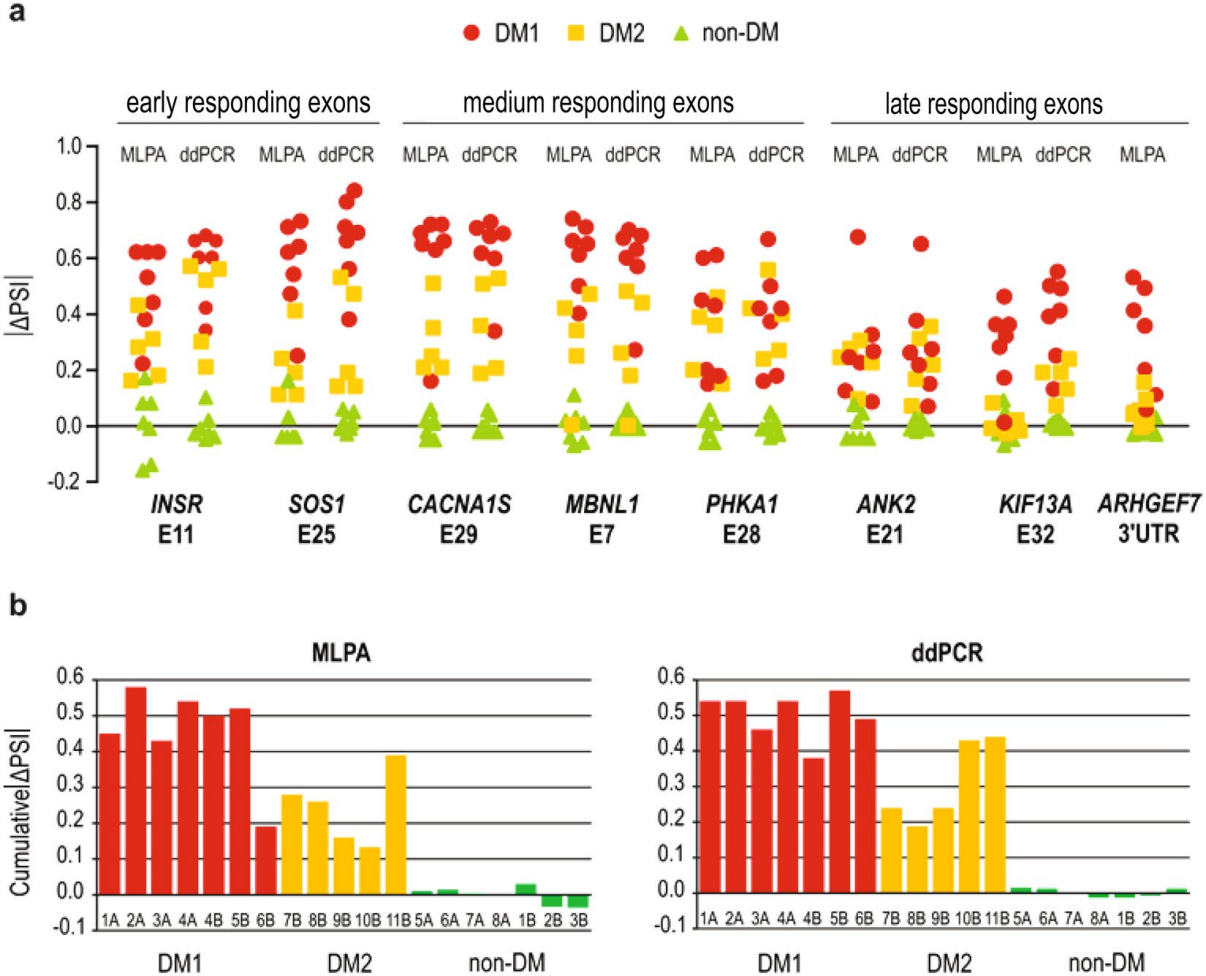
Aberrant Alternative Splicing Analysis with ddPCR and MLPA Assays. (**a**) MLPA and ddPCR results of aberrant alternative splicing for three categories of exons in DM1, DM2 and non-DM samples of human skeletal muscles. For each gene, exon inclusion rate (PSI, the percent of splice in) was calculated and normalized to the average PSI value for non-DM samples, resulting in the delta PSI value for each DM sample. (**b**) Average cumulative results of |ΔPSI| values for DM1, DM2 and non-DM human skeletal muscles (shown in panel a) obtained with MLPA and ddPCR are shown. For two (6B and 10B) out of twelve samples the |ΔPSI| showed some discrepancy between MLPA and ddPCR methods what was correlated with poor RNA quality of these samples.

As illustrated in Fig. 4a, both MLPA and ddPCR methods show strong homogeneity of splicing pattern in the healthy controls, while both DM1 and DM2 samples vary within each exon analyzed. In all DM1 samples, both methods of analysis showed statistically significant differences in comparison to healthy controls (p < 0.0001, for MLPA and ddPCR, Welch’s *t* test). Interestingly, DM2 samples also diverged significantly from the controls, although to a lesser extent (with one exception, *KIF13A* E32, for which MLPA did not show any difference), (p = 0.0064, MLPA; p = 0.0044, ddPCR). For most exons analyzed statistically significant differences were found between DM1 and DM2 samples with both methods, except for *ANK2* E21 and *PHKA1* E28. For all splicing events, there was strong correlation between the results of the MLPA and ddPCR analyses and Pearson correlation coefficient varied between R = 0.628 (p-value = 0.003988) and R = 0.937 (p-value < 1E-05), respectively, for *PHKA1* E28 and for *MBNL1* E7. Average values of cumulative |ΔPSI| (Fig. 4b) confirm different inclusion rates of alternative exons between DM1 and DM2 samples which differ significantly (p = 0.0110, MLPA; p = 0.0204, ddPCR). The sequence of templates binding ddPCR probes was found not to affect the quantification of aberrant alternative splicing as determined with two alternative sets of ex-ON probes for *PHKA1* E28 (Fig. 3a, Supplementary Table S2). Regardless of whether the inclusion probe binds in the alternative exon or at the boundary between exon 27 and 28 of *PHKA1* the results were not statistically different and Pearson correlation coefficient R = 0.990 (p-value < 1E-05) (Supplementary Fig. S1a).

Thus, within each of the methods used there was high reproducibility among independent measurements and for vast majority of samples analyzed both MLPA and ddPCR showed high correlation of data. Dispersion of the variable of at least three replicates for calculated |ΔPSI| expressed as the coefficient of variation (CV) was 0.052 (for MLPA), and 0.072 (for ddPCR). We conclude that both of the methods are equally reliable and sensitive enough to generate accurate and reproducible results when used separately. Thus, we believe that there is no need to use them simultaneously.

### Design of ddPCR Assay for Quantitative Analysis of Absolute Copy Number of DMPK Transcripts

We designed and optimized ddPCR assays for computing the absolute cellular copy number of *DMPK* transcripts. For this purpose, we selected a representative group of DM1 samples derived either from proximal (quadriceps) human skeletal muscles (n = 7) or from proliferating fibroblasts (n = 5) (Supplementary Table S4). All the samples were informative for the rs527221 G > C single nucleotide polymorphism (SNP) located in exon 10 of *DMPK* which in DM1 is linked to its mutant allele (known as *Bpm*I polymorphism)^22^. To distinguish normal and mutant *DMPK* alleles, the FAM and HEX-labeled ZEN probes were designed to bind within the SNP site (Fig. 3b). They had the same nucleotide composition except for the single nucleotide difference (C or G) present in the middle of each probe (Supplementary Table S2). For assay reproducibility and to exclude any potential effect of probe labeling on transcripts quantification we designed two alternative sets of probes (variant 1 shown in Fig. 3b, and variant 2 with reversal of the fluorophores on the 5′-end of ZEN probes) (for details, please see Methods). Supplementary Figure S2 shows selectivity of the FAM and HEX probes designed to recognize the SNP within *DMPK*.

### Quantification of DMPK Transcripts via ddPCR

To characterize cellular abundance of *DMPK* transcripts in DM1 we calculated absolute copy number/cell of normal and mutant *DMPK* mRNAs in proliferating fibroblasts, and determined the fraction of *DMPK*^exp^RNA in skeletal muscles and fibroblast cells.

**Figure 5:**
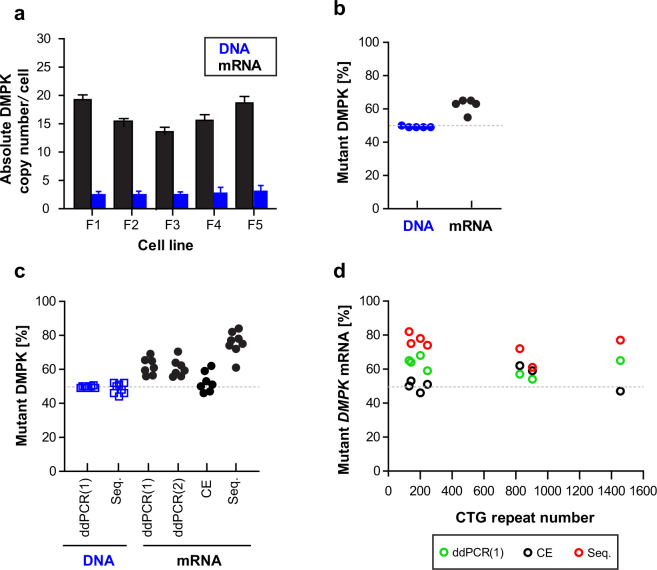
ddPCR-based Quantification of the Absolute DMPK Copy Number per Cell and a Fraction of Mutant DMPK Transcript. (**a**) The absolute *DMPK* copy number per cell in cDNA (black bars) and gDNA (blue bars) from proliferating human DM1 fibroblasts (lines F1-F5). The cells were semi-confluent at the moment of RNA harvest and their passage numbers were between 20 and 37. All the cell lines were immortalized. Bar graphs represent results from at least three independent culture series per line and data are presented as mean ± SD. (**b**) A fraction of mutant *DMPK* transcript and allele in DM1 fibroblasts shown in panel (a). The mean values of independent ddPCR measures performed with *DMPK* probes indicated as variant 1 and 2 are shown. (**c**) A fraction of mutant *DMPK* transcript and allele in, respectively, human DM1 skeletal muscles and peripheral blood. The fraction was determined with: ddPCR (probe variant 1 and 2), capillary electrophoresis (CE) and Sanger sequencing (Seq.). (**d**) The correlation between a fraction of mutant *DMPK* RNA (shown in panel c) and CTG repeats number (~130–1500) in *DMPK* gene is plotted.

Because ddPCR does not directly provide insights into the quantity of RNA molecules per cell, we counted the number of cells prior to harvesting RNA to determine *DMPK* mRNA copy number. Total RNA isolated from one million fibroblast cells was converted to cDNA using random hexamers and subjected to ddPCR with FAM and HEX-labeled probes (Fig. 3b). To test for reverse transcriptase efficiency we used different concentrations of input RNA (125 ng, 250 ng, 500 ng and 1000 ng) in cDNA synthesis (Supplementary Fig. S3). For further analysis we selected samples prepared with 250ng RNA since ddPCR output of *DMPK* mRNA was found to be the highest with this concentration across all fibroblast cell lines used. To validate the accuracy of cell counts we calculated the *DMPK* alleles/cell in gDNA extracted from the aliquots of input cells. Following ddPCR amplification the quantity of *DMPK* alleles was calculated by “equations (1–3)” using the assay output in copies/µl (Supplementary Table S5). As shown in Fig. 5a the number of alleles was about 2 copies per cell in all cell lines analyzed which confirmed the accuracy of cells counts. Two different sets of probes for *DMPK* DNA were used for this calculation and results were not statistically different (p > 0.05) (Supplementary Fig. S4). Next, we computed the number of *DMPK* transcripts/cell using “equations (4–7)” (Supplementary Table S6). As shown in Fig. 5a, the five DM1 fibroblast cell lines had between 15 and 20 molecules of *DMPK* per cell. Because ddPCR probes discriminate between *DMPK*^norm^RNA and *DMPK*^exp^RNA due to the SNP in exon 10, we estimated the proportion of expansion transcripts in the *DMPK* total population. There were between 9 and 11 molecules of *DMPK*^exp^RNA present per cell of DM1 fibroblasts. The proportion of the mutant RNA ranged from 54 to 65% (Fig. 5b). In comparison, there was approximately a one-to-one correspondence between normal and mutant *DMPK* alleles in DNA.

It was previously reported, that normal and mutant *DMPK* transcripts are equally expressed when analyzed in proliferating human DM1 myoblasts^9^. Because our results in fibroblasts indicated otherwise, we set up additional analyses and determined the fraction of *DMPK*^exp^RNA in human DM1 skeletal muscle. As shown in Fig. 5c, the ddPCR results unequivocally indicate the predominance of *DMPK*^exp^RNA (from 53 to 71%) in all DM1 samples tested. We confirmed this result with two other assays including Sanger sequencing and capillary electrophoresis of *Bpm*I digested RT-PCR products (Supplementary Fig. S5). In all gDNA samples from peripheral blood, normal and mutant *DMPK* alleles were about equal which supports our results obtained in gDNA from proliferating fibroblasts (Fig. 5b,c). When analyzing the correlation between the fraction of *DMPK*^exp^RNA in DM1 skeletal muscle and the CTG repeat lengths (assessed from peripheral blood), there was no link between these two variables and the sample from a patient with over 1400 CTG repeats had a comparable fraction of *DMPK*^exp^RNA as those with shorter repeats (Fig. 5d).

In summary, the ddPCR estimated number of cellular *DMPK* mRNA molecules indicates high methodological sensitivity and accurate quantification of low copy targets. High reproducibility of independent measures was indicated by SD ranging from 0.089 to 0.266, whereas strong positive correlation between measures of *DMPK* molecules with probe variant 1 and 2 was shown by Pearson coefficient correlation (R = 0.7508).

### Estimation of Mutant DMPK RNA *in situ* via Fluorescence Hybridization

**Figure 6:**
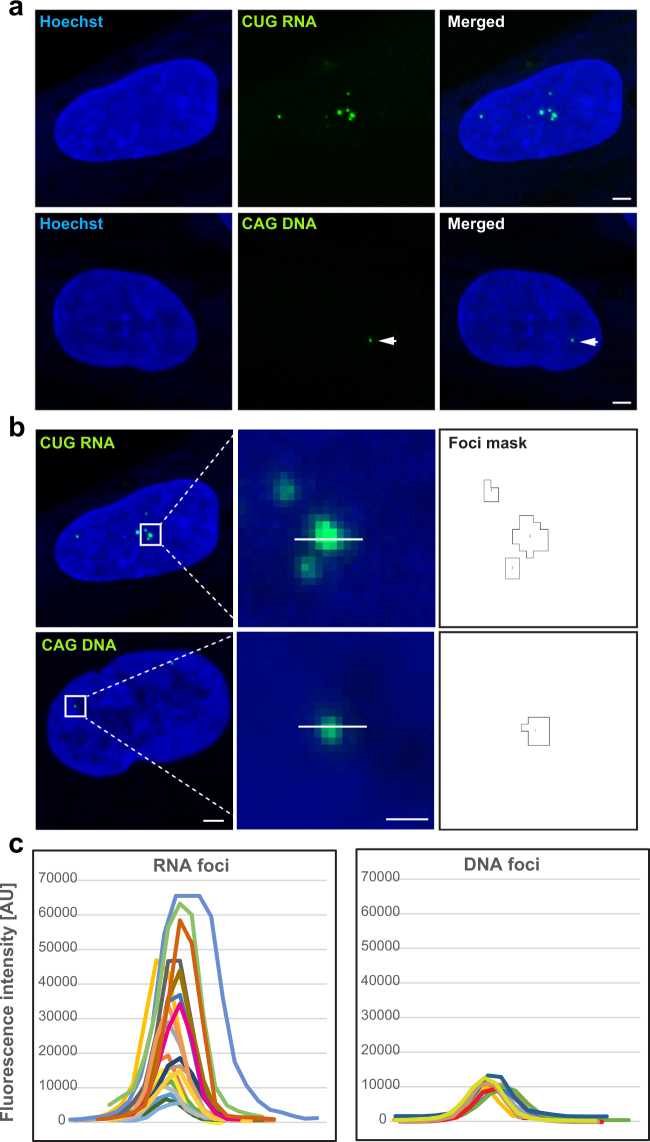
Detection of Mutant CUG Repeat RNA and Mutant CAG Repeat DNA in Nuclear Foci via FISH. (**a**) Representative FISH images of mutant *DMPK* RNA and DNA foci in DM1 human fibroblasts. CUG repeat RNA foci were detected with (CAG)_10_ probe, whereas CAG repeat DNA foci with (CTG)_10_ probe, both labeled with Alexa 488. The nuclear area was identified by Hoechst stain. The arrowheads indicate dim DNA foci. Scale bar, 2 µm. (**b**) 16-bit FISH images (raw data) of RNA and DNA foci and their fluorescence intensity in green channel determined by masks with the outline of recognized foci. Scale bars, 2 µm (left panels) and 0.5 µm (middle panels). (**c**) Maximum values of FISH intensity for RNA and DNA foci from line profiles (as shown in panel b) for randomly selected nuclei of VRS fibroblast cell line. Each plotted color line represents one focus.

**Figure 7:**
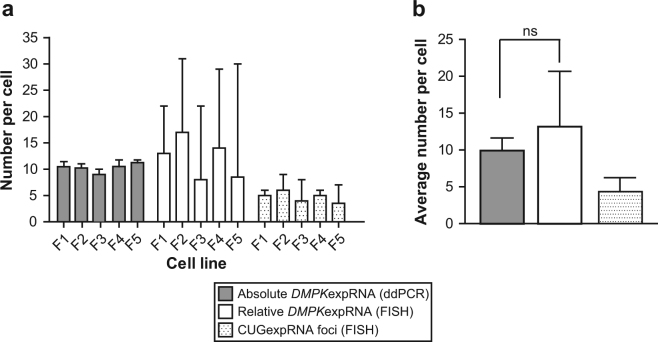
Comparative Analysis of Mutant DMPK mRNA Copy Numbers in Human DM1 Fibroblasts via ddPCR and FISH. (**a**) The absolute numbers of mutant *DMPK* mRNA based on ddPCR results are presented in comparison with FISH estimated relative numbers of the transcript in five DM1 fibroblast cell lines. Plotted are also the numbers of CUG^exp^RNA foci per nucleus. The median values ± SD for each data set are shown. (**b**) The average numbers of mutant *DMPK* RNA and CUG^exp^RNA foci in five DM1 fibroblast cell lines. Data are presented as mean ± SD.

Mutant *DMPK* mRNAs containing the CUG expanded repeats are retained in the nucleus, where they form foci, a molecular hallmark of DM1 pathology^3,23^. It remains an open question, whether foci are composed of multiple aggregating mutant RNAs or whether each focus equals a single RNA molecule. To get an insight into this issue, we applied standard FISH^23^ and compared signals from single molecules of fluorescently labeled probes with signals in repeat expansion DNA (CAG^exp^DNA) and RNA (CUG^exp^RNA) foci. In the five DM1 fibroblasts cell lines (the same as used for *DMPK* quantification via ddPCR assay) there was only one or occasionally two CAG^exp^DNA foci per nucleus, but different numbers of CUG^exp^RNA foci (1–12/nucleus) (Fig. 6a). This result remains in agreement with an earlier report^23^. Measurements of the absolute FISH signal intensity of individual RNA and DNA nuclear foci show that the former represent a very heterogeneous population and higher fluorescence intensity values while signals of the latter were lower and consistent within each group of cells (Fig. 6b,c). To find out whether these features were correlated with different numbers of probes bound in each mutant target (i.e. *DMPK* gene and transcript) or rather with different quantity of the mutant molecules present in these foci, we set up a confocal microscopy analysis and determined fluorescence intensity of single molecules of Alexa 488-labeled probes (Supplementary Figure S6). These values were then used to estimate number of probes in fluorescence signals of DNA and RNA foci (Fig. 6) as well as in random regions outside of the clusters of mutant DMPK (Supplementary Figure S7). As shown in Supplementary Figure S8a the lowest number of Alexa 488 probes was consistently detected in regions outside of nuclear foci (about 1–2 probes) which reflects the probes binding to various targets harboring short CUG and CAG repeats^24^. Significantly higher quantities of probes were estimated in nuclear DNA and RNA foci of all DM1 fibroblasts (Supplementary Figure S8a), however on average there were from two to four times more probes detected in RNA foci. Of note, within each DM1 cell line there was a prominent homogeneity in terms of number of probes in different DNA foci while CUG^exp^RNA foci were more diverse for this feature (Supplementary Figure S8a). Eventually, we computed the relative number of CUG^exp^RNA per nuclear RNA focus (Supplementary Figure S8b) and per nucleus (Supplementary Figure S8c) using median values of DNA foci for each cell line. This estimation of the number of mutant *DMPK* transcripts *in situ* was based on the notion that in diploid DM1 fibroblasts, which are heterozygous for the CTG repeat mutation, each CAG^exp^DNA focus contains one copy of the mutant allele. If we assume that intensity of FISH signal measured for a given DNA focus reflects the presence of one molecule, it should be possible to compute the relative number of expanded CUG repeat *DMPK* transcripts in RNA foci. However, it was taken into account that in an *in situ* hybridization the incorporation of probes used to label RNA or DNA molecules is most likely stochastic process. As shown in Supplementary Figure S8b, the median numbers of mutant CUG repeat transcripts per RNA foci varied between 2 and 4. In each cell line, foci of single CUG^exp^RNA molecules remain in the minority (10–20%) and co-exist with those composed of two to eight molecules. Interestingly, for a subset of foci (5–10%) there appeared to be less than one molecule of mutant RNA per focus. The relative number of CUG^exp^RNA molecules per focus was used to calculate their amount per nucleus. As illustrated in Supplementary Figure S8c, nuclei of each cell line varied in their numbers of CUG^exp^RNA and contained from one to over two dozen of the molecules. While there were on average 4 to 6 RNA foci per nucleus in the fibroblast cells, the estimated median numbers of mutant transcripts ranged from 8 to 17 (Fig. 7a). We found the relative quantities of CUG^exp^RNA per nucleus calculated via FISH not to be significantly different from the absolute values estimated by ddPCR (Fig. 7b).

## Discussion

The selection of the most reliable methods for counting RNA molecules is of paramount importance for understanding the mechanism of pathogenesis of non-coding repeat expansion disorders such as DM1 and DM2^3^. Here, we demonstrate that two quantitative methods, MLPA and ddPCR, can be successfully applied to accurately measure RNA biomarkers of DM pathogenesis which are mechanistically very informative.

Aberrant alternative splicing is a key feature of DM1 and DM2 pathogenesis^4,5^. In DM adult skeletal muscle abnormal expression of embryonic splicing isoforms has been reported for a number of genes^4^. The timing of appearance of certain mis-spliced events correlates well with disease severity measured as a loss of muscle strength^4^ which makes them very useful biomarkers. This hallmark of DM pathogenesis is most informative when the efficacy of investigated compounds is measured to demonstrate their therapeutic value^25–27^. Thus far, profiling of aberrantly spliced transcripts has been performed with high-throughput methods such as specific oligonucleotide-based microarrays and next-generation sequencing, or with traditional RT-PCR and agarose gel quantification of PCR products. Neither method provides both highly reproducible and low cost analyses. We show that MLPA and ddPCR overcome this problem. Our results demonstrate a robust and reliable quantification alternative to calculate splicing isoforms of many genes that are a hallmark of different stages of muscle deterioration in DM.

Mutant *DMPK* mRNAs containing the expanded CUG repeats are retained in the nucleus as distinct foci^3,23,28,29^. While the vast majority of published data have documented the presence and number of CUG foci via FISH (reviewed in^3^) there is still a gap in precise determination of the quantity of *DMPK*^exp^RNA per cell and per nuclear RNA focus. To tackle this issue, we used ddPCR which is an absolute quantification method and we determined *DMPK* copy number in a well-defined quantity of input cells in several human DM1 fibroblast cell lines. In a similar study by Gudde *et al*.^9^, *DMPK* mRNA molecules were calculated in proliferating human DM1 myoblasts using Northern blotting, qPCR and RNA-seq data. Although both the studies indicate a low abundance of *DMPK* transcripts, higher numbers were found in myoblast cells (respectively, 15–20 and 2–50 *DMPK* RNA). Similarly, in human fibroblasts from frontotemporal degeneration (FTD) and amyotrophic lateral sclerosis (ALS) (FTD/ALS)^30,31^, the absolute copy number of *c9orf72* mRNA was estimated at about 15 molecules/cell when measured by ddPCR and qPCR^32^. Interestingly, the intronic part of the transcript containing expanded GGGGCC repeats found in nuclear RNA foci in patients’ cells was detected in only single copy/cell. Thus, in FTD/ALS fibroblasts it appears the mutation-containing intron is degraded more rapidly than the mature transcript is processed. Our ddPCR data suggest that in DM1, the CUG mutation-harboring *DMPK* transcript is turned over slower than its normal-sized counterpart. In patients’ cells and skeletal muscles we consistently detected a higher number of copies of *DMPK*^exp^RNA reaching up to 70% of its entire population. However, except for the differences in RNA processing (i.e. maturation and degradation) variations in expression levels of mutant *DMPK* transcript may also be attributed to differences in RNA synthesis rate as a result of repeat-related chromatin effects.

In DM1, *in situ* hybridization has mostly been used to determine CUG RNA foci numbers^3^. A further application of the technique for more detailed characterization of foci is rather limited due to random incorporation of probes and a lack of tools allowing precise quantification of hybridized molecules. Nonetheless, FISH remains the only method to look at deposits of mutant molecules of *DMPK* transcripts in their natural environment. Here, we applied this technique to get an insight into a problem about quantity of *DMPK* mutant transcripts in nuclear CUG RNA foci. Our estimations based on normalization of fluorescence signals of RNA foci using values of single molecules of Alexa 488 probes led to the conclusion that in DM1 proliferating fibroblasts the vast majority of foci (~80%) consists of a few molecules of mutant *DMPK* transcripts. Importantly, FISH-estimated quantity of *DMPK*^exp^RNA per cell was convergent with what we computed based on ddPCR absolute quantification (Fig. 7b).

Clearly there are important caveats to this work, most notably variability of repeat length estimates and the relationship of probe hybridization to DNA and RNA in RNA FISH experiments. Repeat expansions are highly variable in length and heterogeneous within individuals. Repeat length variability is also manifested in cultures of patient cells. Thus, it is important to point out that the size estimates for repeat lengths in this study are based on the most prominent size of repeat detected. It will not be a uniform length in all cells. In terms of RNA FISH an underlying assumption of our analysis is that the probes hybridize similarly to DNA and RNA. If the probe is binding less well to RNA than to DNA our transcript numbers will be underestimated. Conversely if the CAG probe binds less well to DNA than to RNA our transcript numbers will be overestimated. Furthermore, it is not surprising that we do not see a strict relationship of fluorescence intensity and estimated repeat length in these experiments. In addition to the difficulties estimating precise repeat length in each cell, repeat expansion foci are dynamic structures, continually turning over within cells to reflect the balance between transcription and degradation. Thus, fluorescence intensity will vary accordingly. Nevertheless, our results obtained with two quantitative methods, MLPA and ddPCR, introduce a new important value to the research on DM1 pathogenesis. Knowledge of the precise number of disease-causing RNA molecules could have a broad implication in helping define the limits of the number of MBNL proteins that are sequestered by the expanded repeats and thus the extent of aberrant alternative splicing which results from the depletion.

## Methods

### Samples Used

RNA samples from human skeletal muscles of DM1, DM2 and non-DM were used for splicing analysis (Supplementary Table S3). For *DMPK* allele and transcript quantification lymphocyte DNA and quadriceps RNA samples originated from the same DM1 patients. In addition, human DM1 fibroblasts (immortalized) (Supplementary Table S4) were used and were grown in Dulbecco’s modified eagles medium (DMEM) with penicillin and streptomycin, and 10% fetal calf serum (FCS) (Sigma). The samples, experimental protocols and methods reported in this study were carried out in accordance with the approval of the local ethics committee (NRESCommittee.EastMidlands-Nottingham2). Informed consent was obtained from all subjects.

### Reverse Transcription

Total RNA was extracted with TRIzol (Invitrogen) according to manufacturer’s instruction. Extracted RNA was treated with DNase I (Invitrogen) at 37 °C for 30 min followed by enzyme inactivation at RT for 10 min. Reverse transcription reactions were performed using a SuperScript® III First-Strand Synthesis System (Invitrogen). Briefly, after DNase I treatment, random primers and dNTPs were added to 1 µg of RNA to make volume of 13 µl and heated at 65 °C for 5 min. Then 7 µl of RT mixture (containing 4 µl 5× RT buffer, 1 µl of 0.1 M DTT, 1 µl of RNase OUT, 1 µl of SuperScript® III) was added and RT reaction was performed at 50 °C for 50 min followed by enzyme inactivation at 70 °C for 10 min. cDNA synthesis (as RT+) was accompanied by RT- controls lacking the reverse transcriptase. GoTaq Flexi DNA Polymerase (Promega) and 0.1 mM dNTP mix, 1.5 mM MgCl_2_, and 0.8 μM of each primer were used for any RT-PCR assays to validate primers specificity. To account for any differences in the efficiency of reverse transcriptase in cDNA synthesis, the templates for *DMPK* mRNA quantification were prepared with different concentrations of input RNA i.e. 125 ng, 250 ng, 500 ng and 1000 ng.

### MLPA

MLPA analysis was performed using the in-house designed assays. The probe-set layout was designed according to a previously validated strategy^14,15^. As opposed to commercial tests, this strategy exclusively utilizes short oligonucleotide probes. Each probe consists of two half-probes (each 3′half-probe was phosphorylated at the 5′-end to enable ligation with its sister half-probe); between-probe distance equals 3–5 bp; and the total probe length ranges from 93 to 128 nt. The sequences and detailed characteristics of all probes are shown in Supplementary Table S1. The MLPA probes were synthesized by IDT (Holland). All reagents except for probe-set mix were purchased from MRC-Holland, Amsterdam, Netherlands. The MLPA reactions were run according to the manufacturer’s recommendations and as previously described^11,14^ using 50× diluted cDNA samples and a mixture of 1 nM half-probe oligonucleotides. The products of the MLPA reactions were then diluted 20× in HiDi formamide containing GS Liz600, which was used as a DNA sizing standard, and were separated by size with capillary electrophoresis (POP7 polymer; ABI Prism 3130XL apparatus; Applied Biosystems, Carlsbad, CA, USA). The electropherograms were analyzed using GeneMarker software (version 2.2.0; SoftGenetics, State College, PA, USA). The fluorescent signal of each probe was normalized against the average signal of control probes and then the ratios of signals for each pair of exon-specific probes representing alternative exon inclusion in analyzed samples were calculated.

### ddPCR

Primers and probes used in ddPCR assays were manually designed and synthetized in Integrated DNA Technologies, Inc. (IDT, Belgium). Sequences of primers and probes are listed in Supplementary Table S2. All reactions were prepared using BioRad reagents and assays performed with BioRad equipment. After reverse transcription, ddPCR reaction solution was prepared to a final volume of 25 μl containing 1× ddPCR supermix for probes, 250 nM gene specific primers, 125 nM probes (for ex-ON and ex-OFF), and cDNA (diluted from 20× to 40×). No template control and no reverse transcriptase control (RT-) were included in each ddPCR run to detect possible contaminations. Then, the ddPCR reactions were loaded to a DG8 cartridge and along with 70 μl of droplet generation oil were used to form droplets in a QX100 droplet generator. 40 μl of partitioned emulsion containing droplets was then slowly transferred to 96-Well twin.tec™ Semi-Skirted PCR Plate (Eppendorf). After heat-sealing with foil, the plate containing the droplets was PCR cycled to the final point under conditions at 95 °C, 10 min, 95 °C 30 s and 60 °C for 60 s for 40 cycles, 98 °C for 10 min, then held at 4 °C (for details about annealing temperature for each gene, please see Supplementary Table S2). Following PCR, samples were read on a droplet reader which automatically reads the droplets from each well of the plate. Finally, data were analyzed using QuantaSoft software to determine the number of positive droplets. Instead of using auto-analysis after data acquisition, a manual selection of “+/−,” “−/+,” “+/+” and “−/−” counts was done using the Lasso function in the 2-D plots. The counts were then used by the software to calculate the copy numbers of FAM-positive droplets, HEX-positive, FAM- and HEX-double positive and negative droplets in the four quadrants. For each ddPCR assay serial dilutions of cDNA were used to obtain the lowest number of double-positive droplets; annealing temperature gradients were ran to optimize PCR conditions and to determine the best separation between negative and positive reactions (Supplementary Fig. S1b). Two different sets of probes for measurements of ex-ON transcripts in splicing assay were designed. The target sequence for the first set of probe was the alternative exon, whereas the second type of ex-ON probe was annealing at the boundary of alternative exon and its preceding exon (Fig. 3a). Also, for *DMPK* quantifications two alternative sets of probes were designed (variant 1 shown in Fig. 3b, and variant 2 with reversal of the fluorophores on the 5′-end of ZEN probes) in order to exclude any potential effect of probe labeling on quantifications.

### Quantification of Splicing Isoforms

MLPA and ddPCR results of splicing assays for selected genes were used to compare “percent spliced in” (PSI) values of alternatively spliced cassette exons between non-DM and DM1 as well between non-DM and DM2 (changes in splicing denoted as |ΔPSI|).

### Determination of Absolute Copy Number of *DMPK* per Cell in Genomic DNA and Total RNA from Human Fibroblasts

Cultured human fibroblasts were detached by trypsin solution and cell pellet was washed with ice cold PBS. Then, cell numbers were counted for at least three times by hemocytometer and obtained cell numbers were averaged and then divided into aliquots containing on average one million cells. For further RNA extraction, cells were mixed with Trizol (Invitrogen), whereas gDNA was column isolated (Qiagen). RNA and DNA concentrations were measured by NanoDrop 2000 Spectrophotometer. 250 ng of total RNA was used for cDNA synthesis as described above. Eventually, gDNA and cDNA from corresponding samples were used in ddPCR assays to quantify concentration of *DMPK* (copies per µl) and converted to copies per cell (for details, please see Supplementary Tables S5 and S6).

### Statistical analysis

All experiments were performed at least three times, and the representative results are shown. The data are presented as the mean or median ± SD (as indicated in Figures Legends).

Statistical significance of results of aberrant alternative splicing was determined by the Welch’s unpaired two-tailed *t* test, whereas significance of *DMPK* quantifications via ddPCR and FISH was determined by unpaired two-tailed *t* test; *P* values of <0.05 were considered to be statistically significant in all tests.

## Electronic supplementary material

Supplementary Information
