# Supplementary material for: Quantitative Methods to Monitor RNA Biomarkers in Myotonic Dystrophy
Source: Sci Rep. 2018 Apr 12;8:5885. doi: 10.1038/s41598-018-24156-x (PMC5897446; doi:10.1038/s41598-018-24156-x)
Supplement: Supplementary file 1 — Supplementary Information [file 41598_2018_24156_MOESM1_ESM.pdf]

# SUPPLEMENTARY INFORMATION

## Quantitative Methods to Monitor RNA Biomarkers in Myotonic Dystrophy

Marzena Wojciechowska[1,2]*, Krzysztof Sobczak[3]*, Piotr Kozlowski[2], Saam Sedehizadeh[1], Agnieszka Wojtkowiak-Szlachcic[3], Karol Czubak[2], Robert Markus[1], Anna Lusakowska[4], Anna Kaminska[4], and J. David Brook[1]

[1] University of Nottingham, Queen's Medical Centre, School of Life Sciences, Nottingham, NG7 2UH, United Kingdom.
[2] Institute of Bioorganic Chemistry, Polish Academy of Sciences, Department of Molecular Genetics, Poznan, 61-704, Poland.
[3] Institute of Molecular Biology and Biotechnology, Adam Mickiewicz University, Department of Gene Expression, Poznan, 60-614, Poland.
[4] Medical University of Warsaw, Department of Neurology, Warsaw, 02-097, Poland.

Marzena Wojciechowska: marzena.wojciechowska@nottingham.ac.uk
Krzysztof Sobczak: ksobczak@wp.pl
Piotr Kozlowski: kozlowp@yahoo.com
Saam Sedehizadeh: saam.sedehizadeh1@nottingham.ac.uk
Agnieszka Wojtkowiak-Szlachcic: a.wojtkowiak86@gmail.com
Karol Czubak: czubak.karol@gmail.com
Robert Markus: robert.markus@nottingham.ac.uk
Anna Lusakowska: alusakowska@wum.edu.pl
Anna Kaminska: amkaminska@wum.edu.pl
J. David Brook: david.brook@nottingham.ac.uk

*These authors contributed equaly to this work

Correspondence should be addressed to: david.brook@nottingham.ac.uk
Correspondance may also be addressed to: marzena.wojciechowska@nottingham.ac.uk

## SUPPLEMENTARY METHODS

### Quantification of Mutant *DMPK* via Sanger Sequencing

Sanger sequencing was performed on ABI Prism 3130 genetic analyzer (Applied Biosystems, Carlsbad, CA, USA) according to the manufacturer's recommendations. Samples of gDNA and cDNA from DM1 patients (Supplementary Tab. S4) and non-DM individuals (reference samples from the HapMap project) were used. All samples were informative for the rs527221 G>C polymorphism in exon 10 of *DMPK*. The heights of peaks corresponding to the G variant and the C variant of the allele of the sequencing products were measured with the use of Mutation Surveyor software (SoftGenetics, State College, PA, USA). Based on the obtained results, the ratio of peak G to peak C (G/C ratio) in each sample was calculated. Next, the G/C ratio obtained for each DM1 sample was compared with the average G/C ratio obtained for reference genomic DNA samples. Since in the healthy controls the fraction of the G allele in genomic DNA is 50%, the comparison of G/C ratio between DM1 and reference samples allowed determining the fraction of the G allele in DM1 samples.

### Quantification of *Bpm*I Digested RT-PCR Products via Capillary Electrophoresis

To distinguish normal and mutant *DMPK* transcripts in DM1 samples informative for the rs527221 G>C SNP in exon 10 of *DMPK* (Supplementary Tab. S4), 1/20 of synthesized cDNA was amplified for 26 PCR cycles with N11 forward primer (located in exon 9) and 133 reverse primer which was FAM-labeled (located in exon 10) (Supplementary Tab. S2). The PCR products were subsequently heated to 95$^{o}$C for 2 min followed by cooling to 4$^{o}$C. Five microliters of the PCR mixtures were digested overnight with 2.5 units of *Bpm*I (New England BioLabs) in a total reaction volume of 20 µl at 37°C. Quantitation of digestion products was performed by fragment analysis on an ABI377 sequencer (DNA Sequencing Core, Nottingham University) followed by Peak Scanner2 examination (software v1.0 Thermo Fisher Scientific).

### DNA and RNA *in situ* Hybridization

DM1 human fibroblast cells (Supplementary Tab. S4) were grown and imaged in MatTeck 35 mm dish, No. 1.5 Coverslip, 7 mm Glass Diameter, Uncoated (P35G-1.5-14-C). FISH was performed as previously described[1]. Briefly, cells were fixed in 4% PFA/PBS and washed three times in PBS. For CUG$^{exp}$RNA foci detection pre-hybridization was performed in 40% formamide and 2xSSC buffer for 10 min at RT followed by hybridization in buffer containing 40% formamide, 2xSSC, 0.02% BSA, 2 mM vanadyl ribonucleoside complex, 0.1 mg/ml salmon sperm DNA, and 2 ng/µl DNA probe $(CAG)_{10}$ labeled at the 5′-end with Alexa 488. For CAG$^{exp}$DNA foci detection concentration of formamide in pre-hybridization buffer was increased to 50% and cells were incubated for 1h at 65$^{o}$C. Hybridization buffer was of the same composition as above except for higher concentration of formamide (50%) and $(CTG)_{10}$ probe which was also labeled at the 5′-end

with Alexa 488. Post-hybridization washing in both cases was done in 30% formamide and 2xSSC for 30 min followed by 1xSSC for the next 30 min.

**Microscopy and Image Analysis**

DNA and RNA foci of random nuclei from DM1 cells were visualized and scanned with the confocal unit of Zeiss Elyra PS1 LSM780 microscope (Nottingham University). C-Apochromat 63x/1.2 W Korr M27 water immersion objective was used and correction collar was set up to achieve optimum contrast (0.17 coverslip thickness). The following settings were used for image scanning: lasers 405nm and 488nm: 1.0%; beam splitters MBS 488 and MBS InVis-405; detectors Ch1: 415–490nm; ChS1: 499-579nm. Slow scan (25.2 µsec pixel dwell time) and 4x averaging was used to reduce the noise, therefore dim foci became detectable. All samples were scanned with the same laser and detector settings. Images were recorded as z-stacks. Maximum image projection and image export was done in batch mode using Zeiss Zen Blue to export raw data of 16 bit images. Image analysis was performed in Fiji. Custom written macros (script to automate image analysis and save results) were employed to detect and analyze the fluorescent intensity of RNA and DNA foci in the green channel. Because intensities of RNA foci were on a wide range, a single threshold method was not suitable for recognition of all the foci. Thus, two macros were used, one for bright (Otsu) and another for dim (Triangle) foci which differed only by the thresholding method. Both macros generated a mask with the outline of the recognized foci, and automatically measured results of foci intensity were saved into text files. Masks were compared to the original images, and if some foci were omitted the image was re-analyzed with the second macro. The results from the two runs were merged and further analyzed in MS Excel.

In order to estimate the number of fluorophores (i.e. Alexa 488-labeld probes) bound in RNA and DNA foci we performed single molecule analysis experiment with the following experimental groups: DM1 fibroblasts with no probe included in hybridization reaction, non-DM1 fibroblasts with (CTG)10 probe, non-DM1 fibroblasts with (CAG)10 probe, DM1 fibroblasts with (CTG)10 probe, and DM1 fibroblasts with (CAG)10 probe. Single molecule samples of Alexa 488-labeled probes were prepared in various dilutions in water and deposited on MatTek glass bottom petri dishes. Spots were left to dry to immobilize the single molecules (evaporating solutions were observed as well to record the deposition of the molecules). Single molecules were investigated with four different microscope settings, two of which are presented in the Supplementary Figure S6. The same setting conditions were applied to capture images of the single molecules and cell nuclei. In order to localize the single molecules in the petri dishes we used the AndorXion DU-897 EM-CCD high sensitivity camera (set to 100ms exposure and gain 200). Confocal images were recorded with 1% and 16% laser power in the same focal plane.

Images of single molecules were analyzed with the same macro used to detect the dim foci. To avoid oversampling of high intensity noise pixels, a noise filtering and size restriction was applied (please, see detection mask in Supplementary Figure S6), thus recognition of single

molecules became accurate. The mask was compared with the images scanned at 16% laser and with images taken with the EM-CCD camera. Fluorescent intensity of over a thousand of single molecules from 10 different images was analyzed resulting in a median value of 353. A background subtraction (a value of 222 estimated to be a camera noise and determined in cells with no probe) was applied and single molecule fluorescent intensity was eventually calculated as 131. To calculate the number of fluorescent molecules bound in the nuclear foci we used the following formula:

*Number of probes per focus = (fluorescent intensity of focus – background noise) / (fluorescent intensity of single molecules – background noise).*

In addition to single molecule analysis in foci, we also performed similar calculation in nuclei from each experimental group however in regions outside foci (please, see Supplementary Figure S7). The same background subtraction was applied to the values obtained for these regions. All images were recorded at 16 bit, containing 65535 shades of gray therefore, subtle differences between the background and single molecules were quantifiable. *Technical note:* at 16% laser intensity, CUG RNA foci of DM1 cells became overexposed what made their analysis impossible (please, see Supplementary Figure S6). However, reducing the confocal laser to 1% allowed their analysis along with analysis of still visible the single molecules.

**Southern Blot Determination of CTG Repeat Length in DNA Blood Samples from DM1 Patients**

The diagnosis of DM1 was confirmed by molecular genetic testing and the CTG repeat length estimated using long range PCR and Southern blotting as described previously[2].

## SUPPLEMENTARY FIGURES

### Supplementary Figure S1.

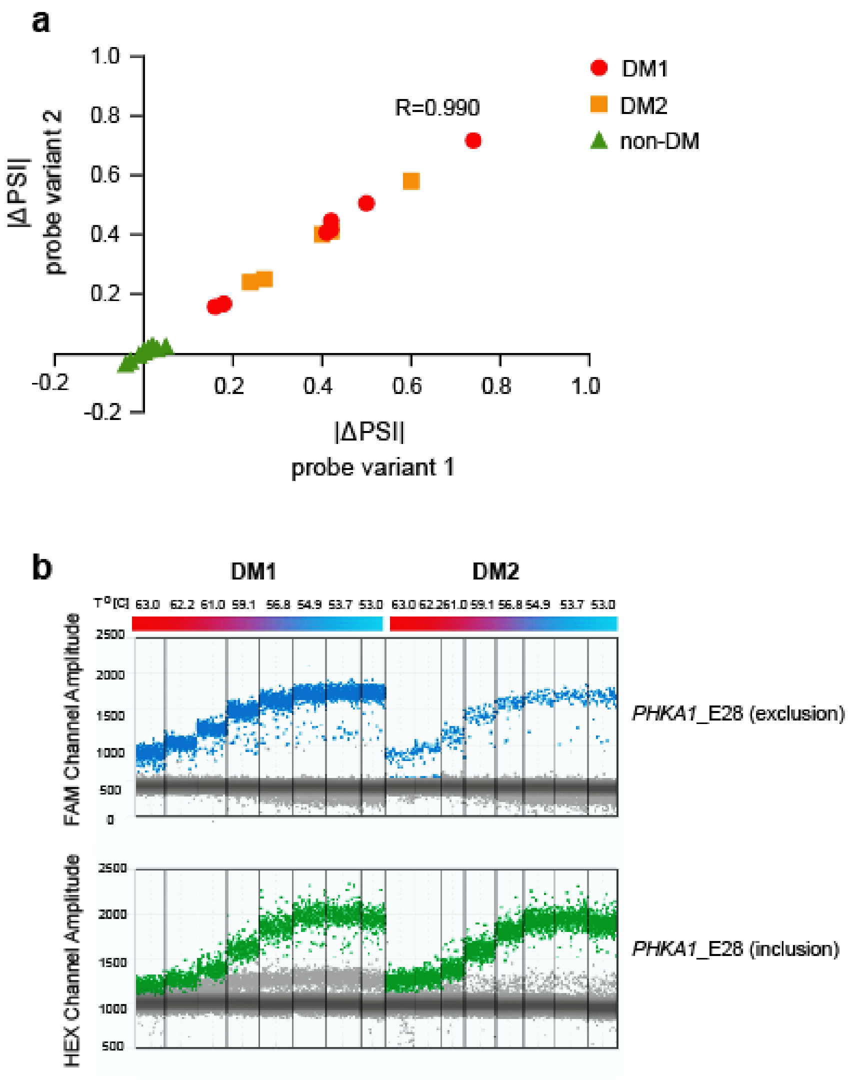


**Supplementary Figure S1.** ***Aberrant Alternative Splicing of PHKA1 Exon 28.***

**(a)** Results of aberrant alternative splicing for *PHKA1* exon 28 with two different variants of ex-ON probes (for details, please see Methods). Sequence of template for binding of ddPCR probes did not affect quantifications of aberrant alternative splicing for *PHKA1* E28. Regardless of whether the inclusion probe binds in the alternative exon (variant 1) or at the boundary between exon 27 and 28 (variant 2) of *PHKA1* the obtained results were not statistically different and Pearson correlation coefficient R=0.990. **(b)** 1-D amplitude plots of FAM- and HEX-labeled products for *PHKA1* exon 28 aberrant alternative splicing in DM1 and DM2 samples from skeletal muscle. Optimization of ddPCR reaction was performed in annealing temperature gradients to determine the best separation between negative and positive droplets.

**Supplementary Figure S2.**

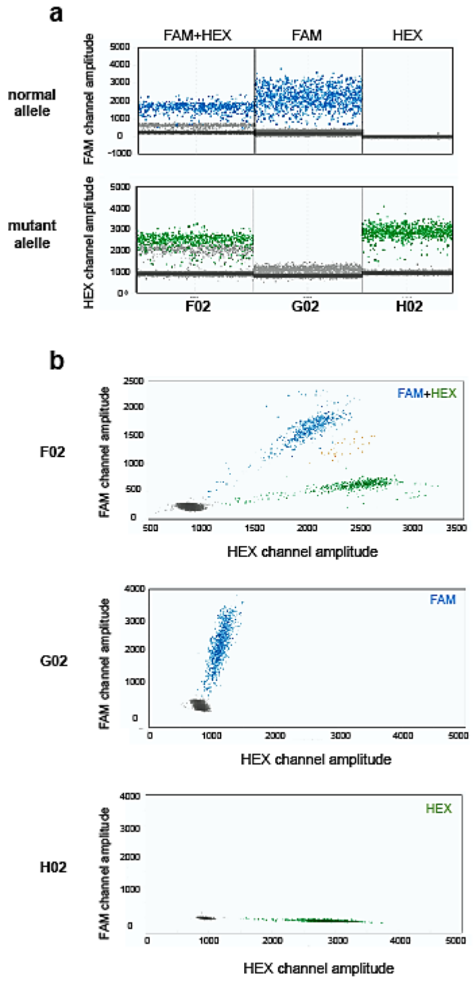


**c**

| Well | Sample | Target | Conc(copies/µL) | |
|---|---|---|---|---|
| F02 | KB (B)_DNA | FAM | 53 | FAM+HEX |
| F02 | KB (B)_DNA | HEX | 59.6 | |
| G02 | KB (B)_DNA_FAM only | FAM | 107 | FAM |
| G02 | KB (B)_DNA_FAM only | HEX | 0 | |
| H02 | KB (B)_DNA_HEX only | FAM | No Call | HEX |
| H02 | KB (B)_DNA_HEX only | HEX | 97.9 | |

**Supplementary Figure S2.** ***Discrimination Between Normal and Mutant DMPK Alleles with Dual-Quencher Probes Used in ddPCR.***

**(a)** 1-D amplitude plots of FAM-labeled (blue) normal *DMPK* assay results (well G02), HEX-labeled (green) mutant *DMPK* assay results (well H02) and a duplex-probe experiment (well F02) of the rs527221 G>C polymorphic DM1 sample of genomic DNA. **(b)** 2-D amplitude plots of ddPCR products shown in panel (a); no double-positive droplets (or any spray into the double-positive region) is detected in either normal (middle panel) or mutant (lower panel) wells. Between blue and green clusters from a duplex experiment (upper panel) with two competitive probes, there are few brown double-positive droplets with both PCR products. Grey cluster, negative droplets with no template. **(c)** ddPCR software calculation of *DMPK* template concentration (copies/µl) from a single probe and duplex-probe experiments shown in (a) and (b).

**Supplementary Figure S3**

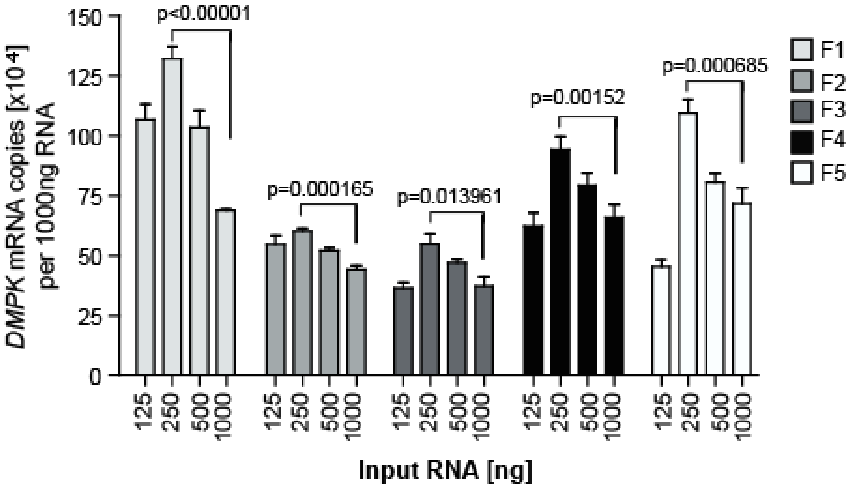


**Supplementary Figure S3*. Measuring the Efficiency of Reverse Transcription via ddPCR for DMPK mRNA.***

The efficiency of reverse transcription (RT) was measured via ddPCR for *DMPK* mRNA after cDNA preparation with the indicated concentrations of input RNA. Total RNA was isolated from semi-confluent DM1 fibroblasts (lines F1, F2, F3, F4 and F5) and used for cDNA synthesis using Super Script III (Invitrogen) and random hexamers. Bar graphs represent results from two independent culture series per line for which triplicate data were generated and are shown as mean ±SD. Statistical significance between results for 250ng and 1000ng input RNA was determined by two-tailed *t* test and P values of <0.05 were considered to be statistically significant.

**Supplementary Figure S4**

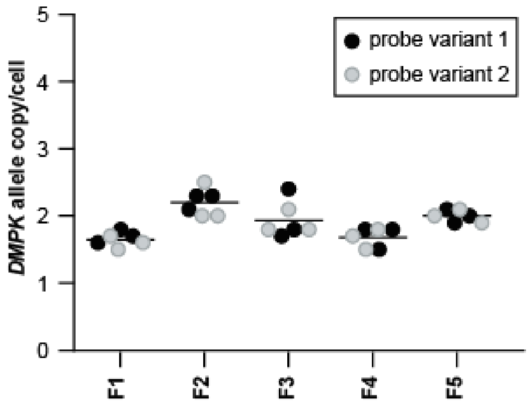


| | | | | | |
|---|---|---|---|---|---|
| Probe variant 1 | SD = 0.100 | 0.115 | 0.300 | 0.173 | 0.100 |
| Probe variant 2 | SD = 0.100 | 0.058 | 0.058 | 0.153 | 0.100 |
| Probe variant 1+2 | SD = 0.105 | 0.200 | 0.266 | 0.147 | 0.089 |

**Supplementary Figure S4.** ***Reproducibility Measures for DMPK Allele Copy Numbers per Cell in Proliferating Human DM1 Fibroblasts.***

ddPCR results of independent analyses display strong inter- and intra-assay agreement in analyzed gDNA samples from DM1 fibroblasts (cell lines F1, F2, F3, F4 and F5). High inter-assay reproducibility (probe variant 1+2) among the six samples was indicated by standard deviation (SD) ranging from 0.089 to 0.266. Similarly, intra-assay standard deviation for the allele's quantities determined separately for probe variant 1 and 2 varied, respectively, from 0.10 to 0.30 and from 0.058 and 0.158.

## Supplementary Figure S5

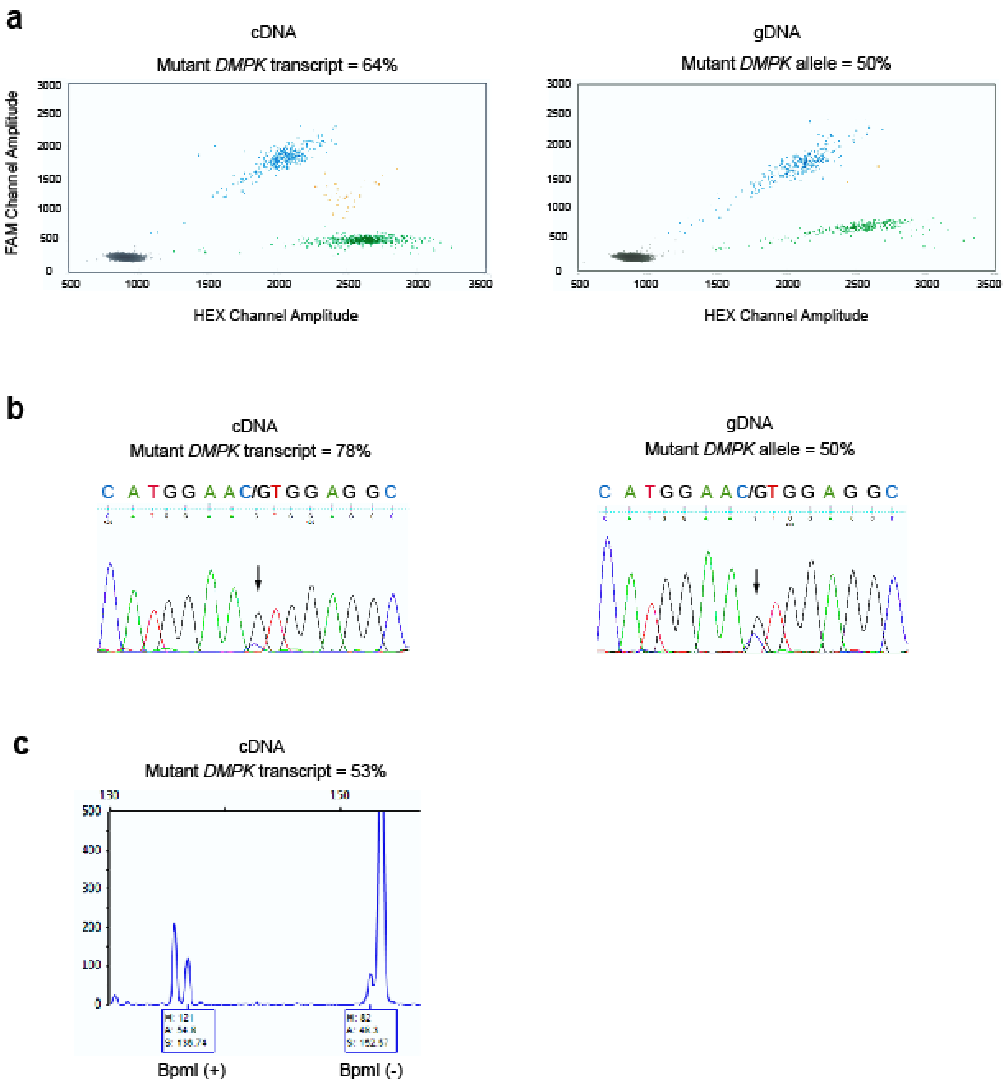


**Supplementary Figure S5.** ***A Fraction of Mutant DMPK Allele and Transcript in Human DM1 Samples Informative for the rs527221 G>C SNP in Exon 10 of DMPK.***

**(a)** Representative 2-D plots displaying droplet populations in separate clusters depending on their fluorescence amplitude following ddPCR for *DMPK* exon 10 in cDNA (quadriceps) (left panel) and corresponding gDNA samples (peripheral blood) (right panel). FAM-positive droplets (blue cluster) with only normal *DMPK* template, HEX-positive droplets (green cluster) with only mutant *DMPK* template from a single ddPCR reaction with one primer pair and two competitive probes (variant 1) are shown. Brown double-positive droplets with both templates are located between the two single-positive clusters. Grey cluster, negative droplets with no template. **(b)** Sanger sequencing results obtained with reverse primer flanking the rs527221 SNP site of *DMPK* in cDNA (left panel) and gDNA (right panel) (for the same samples as used in panel (a). Arrows indicate position of the SNP. **(c)** Capillary electrophoresis of *Bpm*I digested RT-PCR products from cDNA sample (shown in panels a and b).

**Supplementary Figure S6**

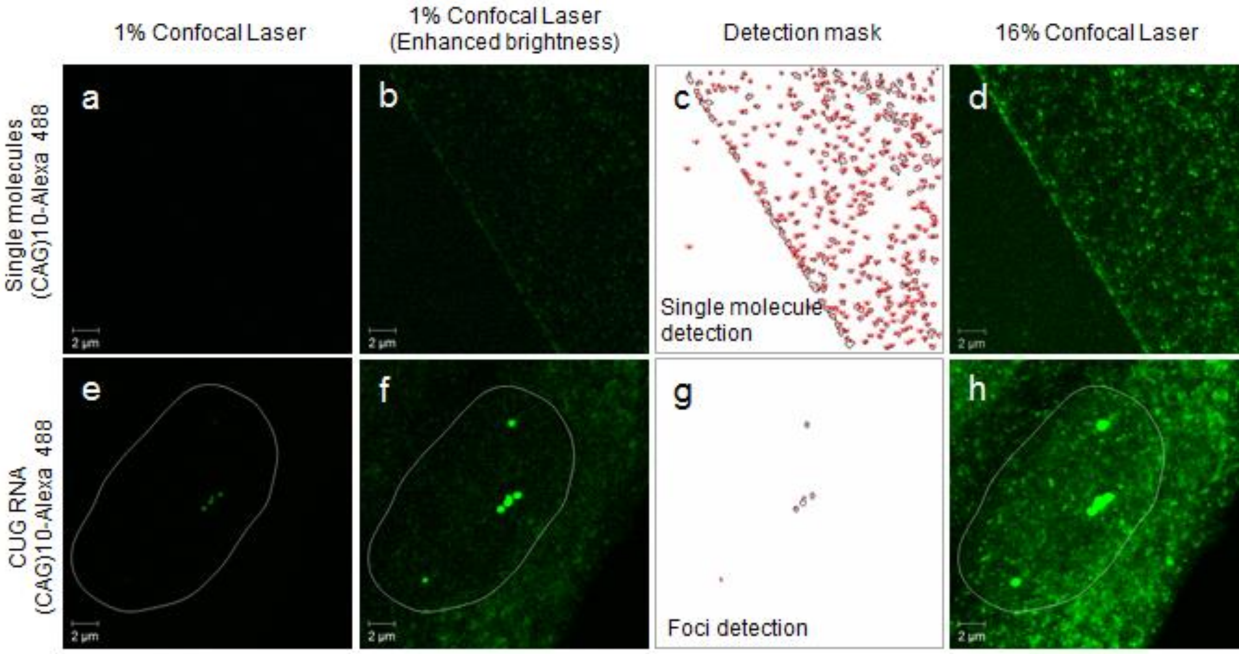


**Supplementary Figure S6.** ***Measuring the Intensity of Single Molecules of Alexa 488-labeled (CAG)10 probes.***

**(a-d)** Confocal images of single molecules of Alexa 488-labeled antisense (CAG)10 probe recorded at 1% and 16% power of confocal laser. Panels (a) and (b) show the same image however brightness of the later was increased to visualize the single molecules. Panel (c) shows a region of interest used for fluorescent intensity analysis of single molecules in image of panel (a) (for technical details please see Supplementary Methods). Panel (d) represents the same field as (a-c) however recorded at 16% confocal laser. **(e-h)** CUG RNA foci in DM1 nucleus (outlined) detected with (CAG)10-Alexa 488 probe are shown with the same laser and visualization settings as the images of (a-d). Scale bars 2µm. *Technical note*: Although the images scanned at 1% laser seem dark, the hidden meaningful data can be extracted. To visualize the data content of the images they are shown at different brightness settings (compare a with b and e with f). Because of the resolution limit of optical microscopy the single molecules appear as spots in the detecting system (3).

**Supplementary FigureS7 (related to Figure S6)**

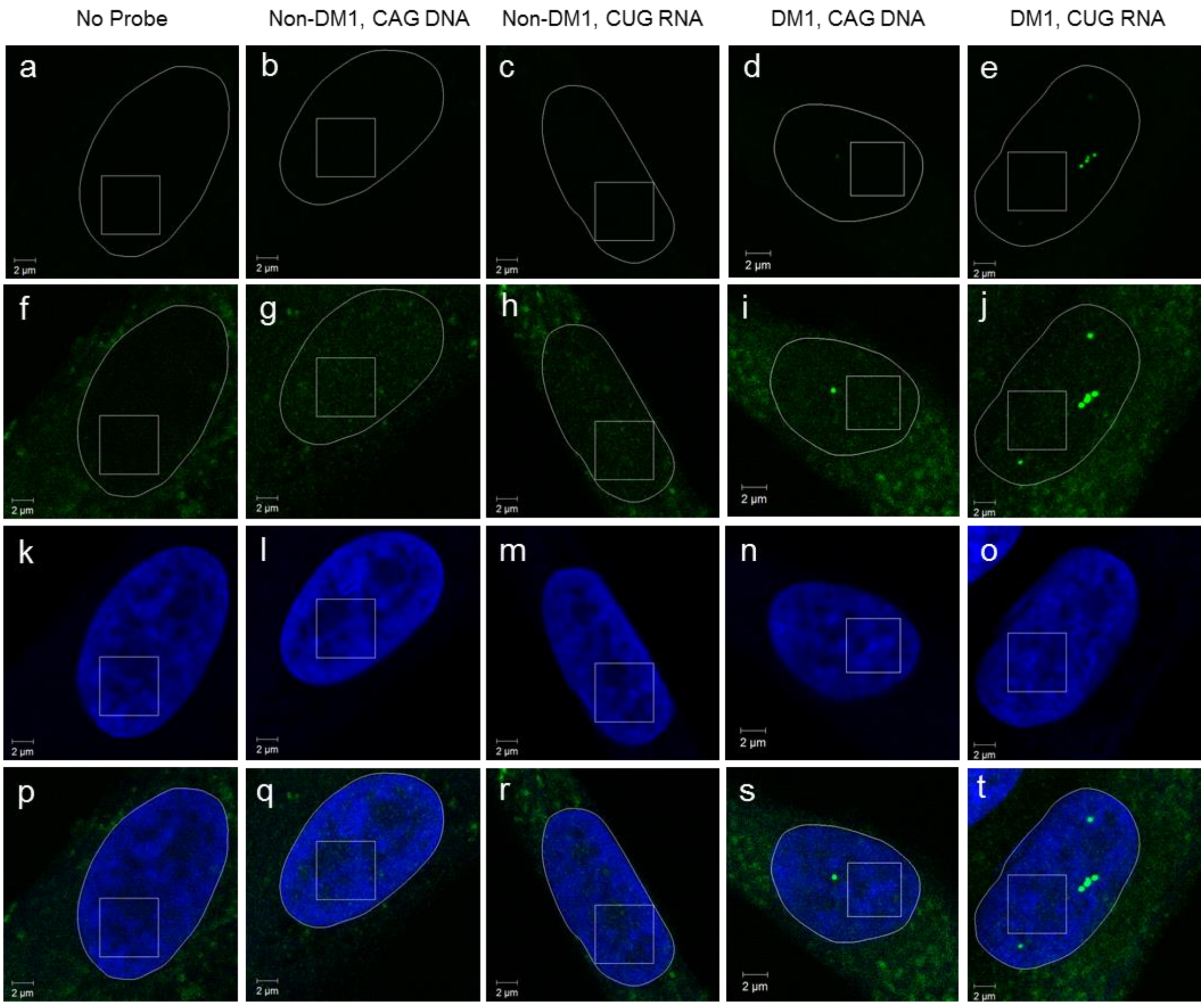


**Supplementary Figure S7.** ***Measuring Single Molecules of Alexa 488-labeled Probes in Random Positions Outside of Foci.***

Selection of rectangular regions of interest (ROI) in nuclei of non-DM1 and DM1 fibroblasts for calculation of fluorescent intensity of Alexa 488-labeled probes in random nuclear positions outside of DNA or RNA foci. The ROIs were subsequently subjected to single molecule analysis (for details, please see Microscopy and Image Analysis). No probe images were analyzed for calculation of camera background noise. Panels (a-e) and (f-j) show corresponding images, respectively, at recorded and enhanced brightness. Panels (k-o) show nuclear DNA staining with Hoechst dye and panels (p-t) are merged images. Panels b and d, images of FISH with (CTG)10-Alexa488 probe; panels c and e, FISH with (CAG)10-Alexa488 probe in hybridization reaction. Scale bars 2µm.

**Supplementary Figure S8 (related to Figure S7 and Figure 6)**

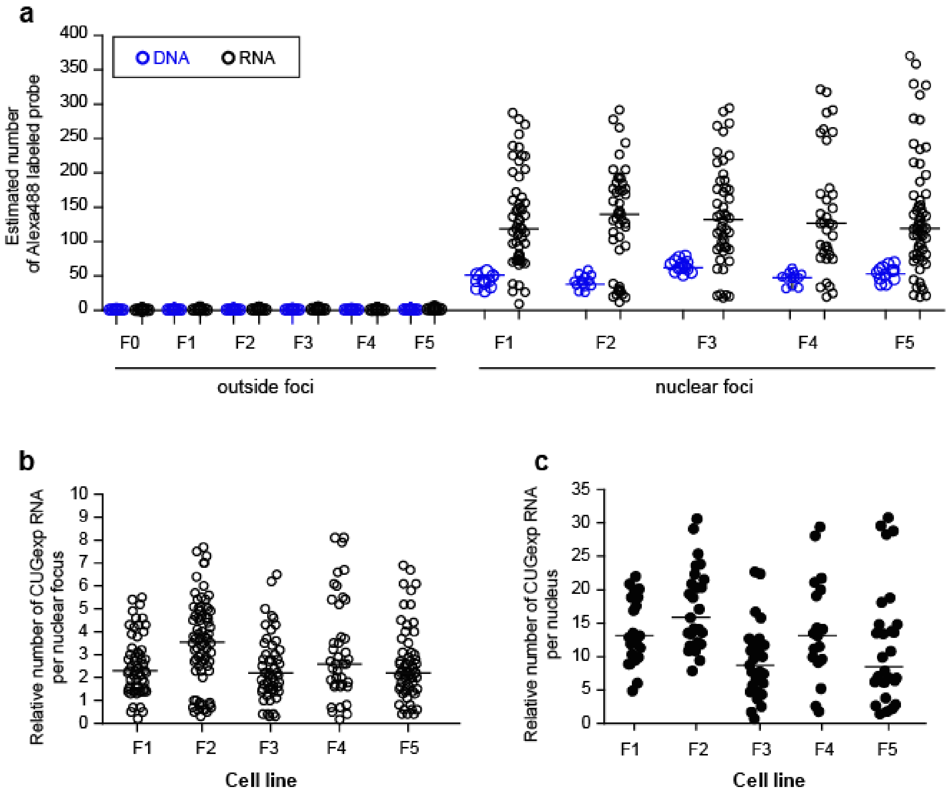


**Supplementary Figure S8.** ***Estimating the Number of Alexa 488-labeled Probes in Nuclear DNA and RNA Foci from Human DM1 Fibroblasts.***

**(a)** Using the microscopy setting presented in Figure S6, the fluorescence intensity of single molecules of Alexa488-labeled probes was determined. Then, intensity of nuclear regions of interest (ROI) outside of DNA or RNA foci (Figure S7) as well as in the nuclear foci (Figure 6) was measured. Obtained values were filtered to remove camera noise. Next, the median value of single molecules of Alexa488-labeled probes was used to calculate quantity of probes outside of nuclear foci as well as in DNA and RNA foci of DM1 fibroblasts (cell lines F1-F5) and in non-DM1 cells (F0). **(b and c)** Using the estimated single probe values for DNA and RNA foci the relative number of expanded CUG repeat *DMPK* transcripts per RNA foci (panel b) and per nucleus (panel c) were calculated. For normalization of values of individual RNA foci the median values of DNA foci (calculated individually for each cell lines) were used. The median values for each data set are shown on the graphs.

## SUPPLEMENTARY TABLES

**Supplementary Table S1.** *Probes and primers used in MLPA assay.*

**Supplementary Table S2.** *Dual-quencher-probes and primers used in ddPCR assays*.

**Supplementary Table S3.** *Samples of human skeletal muscles used in aberrant alternative splicing analysis.*

**Supplementary Table S4.** *Human skeletal muscles and fibroblasts used in DMPK allele and transcript quantification.*

**Supplementary Table S5.** *Equations to calculate DMPK allele copy number per cell.*

**Supplementary Table S6.** *Equations to calculate DMPK transcript copy number per cell.*

**Supplementary Table S1.** ***Probes and primers used in MLPA assays.***

**Supplementary Table S1. Probes and primers used in MLPA assays.**

| probes A | probes B | probes C |
|---|---|---|
| Primer-specific sequences (PSSs) | Primer-specific sequences (PSSs) | Primer-specific sequences (PSSs) |
| stuffer sequences (SSs) | stuffer sequences (SSs) | stuffer sequences (SSs) |
| target-specific sequences (TSSs) | target-specific sequences (TSSs) | target-specific sequences (TSSs) |

| 5' half probe | | | | | 3' half probe | | | |
|---|---|---|---|---|---|---|---|---|
| ID | Sequence 5'-3' | Modification | Probe length [bp] | Product length [bp] | ID | Sequence 5'-3' | Modification | Probe length [bp] |
| ANK2 A | GGGTTCCCTAAGGGTTGGAcgctactactattagtagaattgaGACTCGATGAAGATGCAGCTTTTGAAAAG | NO | 72 | A+C=148bp | - | - | - | - |
| ANK2 B | GGGTTCCCTAAGGGTTGGAcgctactactattagtagaattgatgccaccttttcCGCCGGCGAGTGATTATTCAG | NO | 76 | B+C=152bp | ANK2 C | GGAGACGATATGCCTGAAATACCCCatgtatctaatggtcaaactaaatctacTCTAGATTGGATCTTGCTGGCGC | 5'-phosphate | 76 |
| KIF13A A | GGGTTCCCTAAGGGTTGGA cgctactactattagtagaattCAAAGAGGGCTGGACAGTTACCAG | NO | 65 | A+C=130bp | KIF13A C | GAAGAAGACTTAAACTGCGTAAGGGAGAGGaactaaatctacTCTAGATTGGATCTTGCTGGCGC | 5'-phosphate | 65 |
| - | - | - | - | A+B=134bp | KIF13A B | AGAGATGATGAGGATGGTGATGATATGGtggtcaaactaaatctacTCTAGATTGGATCTTGCTGGCGC | 5'-phosphate | 69 |
| SOS1 A | GGGTTCCCTAAGGGTTGGAcgctactactattagtagaattgaCGATCATTCGAGCCCTTTTCACTCAA | NO | 69 | A+B=137bp | SOS1 B | GCAATGATACCGTCTTTATCCAAGTTACTCTGaaactaaatctacTCTAGATTGGATCTTGCTGGCGC | 5'-phosphate | 68 |
| - | - | - | - | A+C=141bp | SOS1 C | GATCTGCTTCTGTATCATCTATAAGTTTAACCAAAGGCactaaatctacTCTAGATTGGATCTTGCTGGCGC | 5'-phosphate | 72 |
| MBNL1 A | GGGTTCCCTAAGGGTTGGAtactactattagtagaattgatgccaccttttcagctcgcgccAGCCACCGCAGCTGCCATG | NO | 81 | A+C=160bp | MBNL1 C | GGAATTCCTCAAGCTGTACTTCCgttattgaccatttgcgaaatgtatctaatggtTCTAGATTGGATCTTGCTGGCGC | 5'-phosphate | 79 |
| - | - | - | - | A+B=164bp | MBNL1 B | ACTCAGTCGGCTGTCAAATCACTGAAtttgcgaaatgtatctaatggtcaaactaaatctTCTAGATTGGATCTTGCTGGCGC | 5'-phosphate | 83 |
| CACNA1S A | GGGTTCCCTAAGGGTTGGAcgctactactattagtagaattgatgccaccttttcagctcgcgCATTGATGTCATCCTCAGTGAGATCGAC | NO | 91 | A+C=180bp | CACNA1S C | GACCCAGATGAGAGTGCCCGttattgaccatttgcgaaatgtatctaatggtcaaactaaatctacTCTAGATTGGATCTTGCTGGCGC | 5'-phosphate | 89 |
| - | - | - | - | A+B=184bp | CACNA1S B | ACTTTCCTGGCCTCCAGCGGcaggttattgaccatttgcgaaatgtatctaatggtcaaactaaatctacTCTAGATTGGATCTTGCTGGCGC | 5'-phosphate | 93 |
| INSR A | GGGTTCCCTAAGGGTTGGAcgctactactattACAACGTGGTTTTCGTCCCCAG | NO | 54 | A+B=105bp | INSR B | AAAAACCTCTTCAGGCACTGGTGCCtacTCTAGATTGGATCTTGCTGGCGC | 5'-phosphate | 51 |
| - | - | - | - | A+C=111bp | INSR C | GCCATCTCGGAAACGCAGGTCaaactaaatctacTCTAGATTGGATCTTGCTGGCGC | 5'-phosphate | 57 |
| PHKA1 A | GGGTTCCCTAAGGGTTGGAcgctactactattagtagaattgatgccaccttttcagGGCATGCAGTTAAAAAGTGAGATAAAGCAG | NO | 87 | A+C=172bp | PHKA1 C | TCACCTGGAACCTCTATGACTCCAAGTttgcgaaatgtatctaatggtcaaactaaatctacTCTAGATTGGATCTTGCTGGCGC | 5'-phosphate | 85 |
| - | - | - | - | A+B=176bp | PHKA1 B | GTGGAATTTCGTAGACTGTCAATCTCAGCatttgcgaaatgtatctaatggtcaaactaaatctacTCTAGATTGGATCTTGCTGGCGC | 5'-phosphate | 89 |
| ARHGEF7 A | GGGTTCCCTAAGGGTTGGAcGCCAAAACAAGGCAAACACTCAATTCAA | NO | 48 | A+B=93bp | ARHGEF7 B | CATGGCAAGGCACTGACCTGATTCTAGATTGGATCTTGCTGGCGC | 5'-phosphate | 45 |
| - | - | - | - | A+C=99bp | ARHGEF7 C | GTTCACGCAAAGAATCTGCTCCACAtacTCTAGATTGGATCTTGCTGGCGC | 5'-phosphate | 51 |
| PKP2 A | GGGTTCCCTAAGGGTTGGAcgctactaCTGAAGCTCGGAAGAGGGTTAACC | NO | 51 | A+C=102bp | PKP2 C | AGCTTCGTGGCATCCTCAAGCTTCctacTCTAGATTGGATCTTGCTGGCGC | 5'-phosphate | 51 |
| DAB2 A | GGGTTCCCTAAGGGTTGGAcgctactactaCTAGGTGGTGTAACTGTCACACTCCCT | NO | 57 | A+C=114bp | DAB2 C | CAGGCAGGACCATGGAACACAaaactaaatctacTCTAGATTGGATCTTGCTGGCGC | 5'-phosphate | 57 |
| IGF1 A | GGGTTCCCTAAGGGTTGGAcgctactactattagtagaattgatgccaccttGGGCTTTTATTTCAACAAGCCCACAG | NO | 78 | A+C=156bp | IGF1 C | GGTATGGCTCCAGCAGTCGGAtgcgaaatgtatctaatggtcaaactaaatctacTCTAGATTGGATCTTGCTGGCGC | 5'-phosphate | 78 |

*Font colors in the Table corresponds to the probes colors in Figure 2a.*

**Supplementary Table S2.** ***Primers and dual-quencher-probes used in ddPCR assays.***

***Primers Used:***

| Gene Name | Alternative Exon Length [bp] | Sequence [5'-3'] | PCR product length [bp] | Annealing temperature [$^{o}$C] |
|---|---|---|---|---|
| ***ANK2*** | 84 | **F:** CCAGCCTGAGACCTGTGAAA | 174 | 58 |
| | | **R:** CCTTCAGAGGATACATACCGCC | | |
| ***KIF13A*** | 39 | **F:** GTAACTGCCAGGTCCACCAA | 130 | 59 |
| | | **R:** GTGCATCTGACCACCTCTCC | | |
| ***SOS1*** | 45 | **F:** AGTACCACAGATGTTTGCAGTG | 191 | 59 |
| | | **R:** TCTGGTCGTCTTCGTGGAGGAA | | |
| ***MBNL1*** | 54 | **F:** GCCCAATACCAGGTCAACCA | 155 | 57 |
| | | **R:** GGCCTCTTTGGTAATGGGGG | | |
| ***CACNA1S*** | 57 | **F:** CCTGATTGTCATTGGCAGCA | 168 | 59 |
| | | **R:** AGCCTCATGACACGGAACAG | | |
| ***INSR*** | 36 | **F**: CTGCACAACGTGGTTTTCG | 108 | 57 |
| | | **R:** TCACATTCCCAACATCGCCA | | |
| ***PHKA1*** | 39 | **F:** GAACGAACTGGGATCATGCAG | 128 | 58 |
| | | **R:** GCACTAGGAAAGGACCCACT | | |
| ***DMPK*_cDNA_ ddPCR** | - | **F:** TGGGCTACTCCTACTCCTGC | 101 | 60 |
| | | **R:** TTGCACGTGTGGCTCAAG | | |
| ***DMPK*_DNA_ ddPCR** | - | **F:** GTTCTCCACCAGAGAATCAGCATT | 129 | 60 |
| | | **R:** TTGCACGTGTGGCTCAAG | | |
| ***DMPK*_N11_capill. electrophoresis** | - | **F:** CACTGTCGGACATTCGGGAAGGTGC | 154 | 60 |
| ***DMPK*_133_capill. electrophoresis** | | **R:** GCTTGCACGTGTGGCTCAAGCAGCTG | | |
| ***DMPK*_Sanger seq.** | - | **R:** TGTTTCATCCTGTGGGGACA | | |

***Probes Used:***

| Gene Name | Alternative Exon | Sequence | Binding Exon |
|---|---|---|---|
| ***ANK2*_HEX** | 21 | 5- /5HEX/TTT TGA AAA /ZEN/GGG AGA CGATAT GCCTGA A/3IABkFQ/ -3 | Ex21 |
| ***ANK2*_FAM** | | 5- /56-FAM/TGC AGCTTT /ZEN/TGA AAA GGTTACTAG GAA A/3IABkFQ/ -3 | Ex20/22 |
| ***KIF13A*_HEX** | 32 | 5- /5HEX/ACA GTT ACC /ZEN/AGA GAG ATG ATG AGG ATG GT/3IABkFQ/ -3 | Ex32 |
| ***KIF13A*_FAM** | | 5- /56-FAM/ACA GTT ACC /ZEN/AGG AAG AAG ACT TAA ACT G/3IABkFQ/ -3 | Ex31/33 |
| ***SOS1*_HEX** | 25 | 5- /5HEX/TCT GCC CCA /ZEN/TGG CCC AAG ATC TGC TT/3IABkFQ/ -3 | Ex25 |
| ***SOS1*_FAM** | | 5- /56-FAM/AGC CCT TTT /ZEN/CACTCA AGATCT GCT TCT G/3IABkFQ/ -3 | Ex24/26 |
| ***MBNL1*_HEX** | 7 | 5- /5HEX/TGC CAT GAC /ZEN/TCA GTC GGCTGT CAA A/3IABkFQ/ -3 | Ex7 |
| ***MBNL1*_FAM** | | 5- /56-FAM/CGC AGCTGC /ZEN/CAT GGG AAT TCC TCA AG/3IABkFQ/ -3 | Ex6/8 |
| ***CACNA1S*_HEX** | 29 | 5- /5HEX/ATC GAC ACT /ZEN/TTC CTG GCCTCC AG/3IABkFQ/ -3 | Ex29 |
| ***CACNA1S*_FAM** | | 5- /56-FAM/AGT GAG ATC /ZEN/GAC GAC CCA GAT GAG /3IABkFQ/ -3 | Ex28/30 |
| ***INSR*_HEX** | 11 | 5- /5HEX/TCT TCA GGC /ZEN/ACT GGT GCC GAG G/3IABkFQ/ -3 | Ex11 |
| ***INSR*_FAM** | | 5- /56-FAM/TCG TCC CCA /ZEN/GGC CAT CTC GGA AAC /3IABkFQ/ -3 | Ex10/12 |
| ***PHKA1*_HEX (variant 1)** | 28 | 5- /5HEX/TTC GTA GAC /ZEN/TGT CAA TCT CAG CTG/3IABkFQ/ -3 | Ex28 |
| ***PHKA1*_FAM** | | 5- /56-FAM/AGA TAA AGC /ZEN/AGT CAC CTG GAA CC/3IABkFQ/ -3 | Ex27/29 |
| ***PHKA1*_HEX (variant 2)** | | 5- /5HEX/TAA AGC AGG /ZEN/TGG AAT TTC GTA GA/3IABkFQ/ -3 | Ex27/28 |

| | ***Bpm*I polymorphism** | | **Binding allele** |
|---|---|---|---|
| ***DMPK*_HEX (variant 1)** | G | 5- /5HEX/CAC ACC CAT /ZEN/GGA A**G**T GGA GGC CGA GCA /3IABkFQ/ -3 | Mutant allele |
| ***DMPK*_FAM (variant 1)** | C | 5- /56-FAM/CAC ACC CAT /ZEN/GGA A**C**T GGA GGC CGA GCA /3IABkFQ/ -3 | Normal allele |
| ***DMPK*_HEX (variant 2)** | C | 5- /56-HEX/CAC ACC CAT /ZEN/GGA A**C**T GGA GGC CGA GCA /3IABkFQ/ -3 | Normal allele |
| ***DMPK*_FAM (variant 2)** | G | 5- /5FAM/CAC ACC CAT /ZEN/GGA A**G**T GGA GGC CGA GCA /3IABkFQ/ -3 | Mutant allele |

**Supplementary Table S3.** ***Samples of human skeletal muscles used in aberrant alternative splicing analysis.***

| Sample Name/Gender | Sample Type | Sample Source | Age of onset [years] | Age [years] | CTG Repeat Length [bp] |
|---|---|---|---|---|---|
| 1A/ M | DM1 | Tibialis anterior | 26 | 32 | 430 |
| 2A/ F | DM1 | Tibialis anterior | 17 | 41 | 1520 |
| 3A/ F | DM1 | Tibialis anterior | 26 | 53 | 480 |
| 4A/ F | DM1 | Tibialis anterior | 50 | 69 | 270 |
| 4B/ F | DM1 | Quadriceps | 43 | 50 | ND |
| 5B/ F | DM1 | Quadriceps | 29 | 44 | ND |
| 6B/ M | DM1 | Quadriceps | 47 | 55 | ND |
| 7B/ M | DM2 | Quadriceps | 57 | 59 | ND |
| 8B/ F | DM2 | Quadriceps | 54 | 55 | ND |
| 9B/ F | DM2 | Quadriceps | 67 | 70 | ND |
| 10B/ M | DM2 | Biceps branchi | 46 | 46 | ND |
| 11B/ F | DM2 | Biceps branchi | 58 | 74 | ND |
| 5A/ F | Non-DM | Tibialis anterior | - | 65 | <37 |
| 6A/ F | Non-DM | Tibialis anterior | - | 50 | „ |
| 7A/ M | Non-DM | Tibialis anterior | - | 20 | „ |
| 8A/ M | Non-DM | Tibialis anterior | - | 32 | „ |
| 1B/ F | Non-DM | Quadriceps | - | 62 | „ |
| 2B/ F | Non-DM | Quadriceps | - | 35 | „ |
| 3B/F | Non-DM | Quadriceps | - | 52 | „ |

**ND**, not determined

**Supplementary Table S4.** ***Samples used in DMPK allele and transcript quantification.***

| Sample Name/Gender | Sample Type | Sample Source | Age [years] | CTG Repeat Number | *Bpm*I Polymorphism |
|---|---|---|---|---|---|
| DM07.1/M | DM1 | Quadriceps | 43 | 904 | + |
| DM53.1/M | DM1 | Quadriceps | 51 | 143 | + |
| DM01.1/M | DM1 | Quadriceps | 56 | 246 | + |
| DM08.1/F | DM1 | Quadriceps | 43 | 825 | + |
| DM16.1/F | DM1 | Quadriceps | 34 | 130 | + |
| DM17.1/F | DM1 | Quadriceps | 34 | 201 | + |
| DM40.1/F | DM1 | Quadriceps | 46 | 1456 | + |
| | | | | | |
| DM07.1/M | DM1 | Blood | 43 | 904 | + |
| DM53.1/M | DM1 | Blood | 51 | 143 | + |
| DM01.1/M | DM1 | Blood | 56 | 246 | + |
| DM08.1/F | DM1 | Blood | 43 | 825 | + |
| DM16.1/F | DM1 | Blood | 34 | 130 | + |
| DM17.1/F | DM1 | Blood | 34 | 201 | + |
| DM40.1/F | DM1 | Blood | 46 | 1456 | + |
| DM14.1/F | DM1 | Blood | 41 | 50 | + |
| | | | | | |
| F1 | DM1 | Fibroblast | ND | 143 | + |
| F2 | DM1 | Fibroblast | ND | 400 | + |
| F3 | DM1 | Fibroblast | ND | 825 | + |
| F4 | DM1 | Fibroblast | ND | 1456 | + |
| F5 | DM1 | Fibroblast | ND | ND | + |

**ND**, not determined
All the *Bpm*I informative samples originate from unrelated individuals except for DM16.1 and DM17.*1.*

**Supplementary Table S5.** ***Equations to calculate DMPK allele copy number per cell.***

*First, we calculated the total copies per well of ddPCR reaction:*

$$\frac{\textbf{Total copies}}{\textbf{Well}} = \text{Dilution factor} \times \text{concentration (copies/}\mu\text{l)} \times \text{reaction vol. (}\mu\text{l)} \qquad \textbf{equation (1)}$$

*Then, we calculated total copies per whole DNA sample:*

$$\frac{\textbf{Total copies}}{\textbf{Total DNA isolated}} = \frac{\text{total copies}}{\text{well}} \times \text{vol. DNA (}\mu\text{l)} \qquad \textbf{equation (2)}$$

*Then, we normalized to the number of input cell:*

$$\frac{\textbf{Allele}}{\textbf{Cell}} = \frac{\dfrac{\text{total copies}}{\text{DNA isolated}}}{\text{number input cells}} \qquad \textbf{equation (3)}$$

All of the calculations above used the output in copies/µl from the Bio-Rad reader software.

**Supplementary Table S6.** ***Equations to calculate DMPK transcript copy number per cell.***

*First, we calculated the total copies per well of ddPCR reaction:*

$$\frac{\textbf{Total copies}}{\textbf{Well}} = \text{Dilution factor} \times \text{concentration (copies/}\mu\text{l)} \times \text{reaction vol. (}\mu\text{l)} \qquad \textbf{equation (4)}$$

*Then, we calculated total copies per whole cDNA sample:*

$$\frac{\textbf{Total copies}}{\textbf{cDNA = 1ug RNA}} = \frac{\text{total copies}}{\text{well}} \times \text{vol. cDNA (}\mu\text{l)} \qquad \textbf{equation (5)}$$

*Next, we calculated the total copies per whole RNA sample:*

$$\frac{\textbf{RNA copies}}{\textbf{Total RNA isolated}} = \text{copies in 1}\mu\text{g} \times \text{total RNA (}\mu\text{g)} \qquad \textbf{equation (6)}$$

*Then, we normalized to the number of input cell:*

$$\frac{\textbf{RNA}}{\textbf{Cell}} = \frac{\left(\dfrac{\text{total copies}}{\text{RNA isolated}}\right)}{\text{number input cells}} \qquad \textbf{equation (7)}$$

All of the calculations above used the output in copies/µl from the Bio-Rad reader software.

## SUPPLEMENTARY REFERENCES

**1.** Wojciechowska, M., Taylor, K., Sobczak, K., Napierala, M. and Krzyzosiak, W.J. Small molecule kinase inhibitors alleviate different molecular features of myotonic dystrophy type 1. RNA Biol. **11**, 742-754 (2014).

**2.** Kamsteeg, E.J., Kress, W., Catalli, C., Hertz, J.M., Witsch-Baumgartner, M., Buckley, M.F., van Engelen, B.G., Schwartz, M. and Scheffer, H. Best practice guidelines and recommendations on the molecular diagnosis of myotonic dystrophy types 1 and 2. Eur. J. Hum. Genet. **20**, 1203-1208 (2012).

**3.** Betzig E., Patterson G. H., Sougrat R., Lindwasser O. W., Olenych S., Bonifacino J. S., Davidson M. W., Lippincott-Schwartz J., Hess H. F. Imaging Intracellular Fluorescent Proteins at Nanometer Resolution. *Science* ***313**,* 1642-1645 (2006).
